# Phospholipids in lipoproteins: compositional differences across VLDL, LDL, and HDL in pregnant women

**DOI:** 10.1186/s12944-019-0957-z

**Published:** 2019-01-22

**Authors:** Sebastian Rauschert, Antonio Gázquez, Olaf Uhl, Franca F. Kirchberg, Hans Demmelmair, María Ruíz-Palacios, María T. Prieto-Sánchez, José E. Blanco-Carnero, Anibal Nieto, Elvira Larqué, Berthold Koletzko

**Affiliations:** 1 LMU - Ludwig-Maximilians-Universität Munich, Div. Metabolic and Nutritional Medicine, Dr. von Hauner Children’s Hospital, University of Munich Medical Center, 80337 Munich, Germany; 2 Department of Physiology, Faculty of Biology, University of Murcia, Murcia, Spain; 3 Obstetrics and Gynecology Service, Virgen de la Arrixaca Clinical Hospital, University of Murcia, Murcia, Murcia, Spain

**Keywords:** Pregnancy, Sphingolipids, Sphingomyelin, Lipidomics, Obesity, Phosphatidylcholine, Lipoproteins

## Abstract

**Objective:**

The aim of this study was to analyse the differences in the phospholipid composition of very low density (VLDL), low density (LDL) and high density lipoprotein (HDL) monolayers in pregnant lean and obese women.

**Methods:**

LDL, HDL, and VLDL were isolated from plasma samples of 10 lean and 10 obese pregnant women, and their species composition of phosphatidylcholines (PC) and sphingomyelins (SM) was analysed by liquid-chromatography tandem mass-spectrometry. Wilcoxon-Mann-Whitney U test and principal component analysis (PCA) were used to investigate if metabolite profiles differed between the lean/obese group and between lipoprotein species.

**Results:**

No significant differences have been found in the metabolite levels between obese and non-obese pregnant women. The PCA components 1 and 2 separated between LDL, HDL, and VLDL but not between normal weight and obese women. Twelve SM and one PCae were more abundant in LDL than in VLDL. In contrast, four acyl-alkyl-PC and two diacyl-PC were significantly higher in HDL compared to LDL. VLDL and HDL differed in three SM, seven acyl-alkyl-PC and one diacyl-PC (higher values in HDL) and 13 SM (higher in VLDL). We also found associations of some phospholipid species with HDL and LDL cholesterol.

**Conclusion:**

In pregnant women phospholipid composition differs significantly in HDL, LDL and VLDL, similar to previous findings in men and non-pregnant women. Obese and lean pregnant women showed no significant differences in their lipoprotein associated metabolite profile.

**Electronic supplementary material:**

The online version of this article (10.1186/s12944-019-0957-z) contains supplementary material, which is available to authorized users.

## Introduction

Obesity and its comorbidities are a major public health concern [1]. It has been shown that pregnancy and early life can already set the path towards an adverse metabolic state as in obesity. In this regard, maternal obesity during gestation is a risk factor for offspring obesity later in life [2]. Mechanistically, lipoproteins are one of the main sources for fetal nutrient supply, as they contain triacylglycerols (TG). Obesity is associated with a disturbed lipoprotein profile [3]. Hence, higher lipoprotein levels and an altered structure of the phospholipid monolayer are an important object of research, as modifications might have consequences for fetal energy supply and early programming of later adverse health outcomes due to energy over- or undersupply.

In the fasting state, the function of very low density lipoproteins (VLDL) is to transport fatty acids in the form of TG from the liver to extrahepatic tissues and, during pregnancy, to the fetus for energy metabolism and structural maintenance [4]. VLDL particles are converted into low density lipoproteins (LDL) that contain less TG and more cholesterol than VLDL. LDL cholesterol can be taken up by tissue mediated by the LDL receptor, or at high levels be deposited in scavenger cells in the intima-media of blood vessels which contributes to cardiovascular disease risk [5]. In contrast, HDL recollects cholesterol from extrahepatic tissues and transports it back to the liver for excretion with bile, contributing to reducing cardiovascular risk [6].

Total plasma fatty acids are strongly associated with lipoprotein metabolism and various lipoprotein features, such as cholesterol and content of saturated species are related with cardiovascular health [7–9]. Despite existing evidence of the importance of lipoprotein fatty acid composition for the metabolism and functionality of some of these particles [10], differences in molecular species found in different lipoproteins have hardly been studied.

The surface of all lipoproteins is comprised of phospholipids, mainly phosphatidylcholine (PC) and sphingomyelin (SM) [11]. Those two groups of phospholipids have been positively associated with higher BMI in clinical targeted metabolomics studies [12–15]. Most of the current metabolomics studies use whole plasma for blood fatty acid and phospholipid analyses [16] and usually different lipoproteins are not further separated before analysis. Thus, differences of the lipoprotein composition are not considered.

Differences in the phospholipid composition of lipoproteins have previously been shown in males and non-pregnant females [17].

We aimed to characterize the phospholipid composition of LDL, HDL and VLDL in obese and lean pregnant women, which, apart from lipoprotein species in pregnancy in general [18], so far has not been described in detail. Circulating lipids change considerably during pregnancy and lipid levels have been associated with offspring anthropometry [19]. High placental expression of endothelial lipase (EL), which preferentially cleaves phospholipids, indicates involvement of phospholipids in placental transfer of fatty acids [20]. Thus, a comparison of phospholipids between lipoproteins in pregnant women can improve the understanding of lipid metabolism during pregnancy and supports the planning of further metabolomics studies in respect to the inclusion of lipoprotein specific analyses.

## Materials and methods

### Subjects

Twenty pregnant women were studied at the time of parturition (between 37 and 41 weeks of gestation) in the Obstetrics and Gynecology Service of the Virgen de la Arrixaca Clinical Hospital, Murcia (Spain), including 10 obese women (pre-pregnancy BMI > 30 kg/m^2^) and 10 women with normal weight (pre-pregnancy BMI 20-25 kg/m^2^). All women satisfied the following inclusion criteria: singleton pregnancy, term delivery, age 18–40 years, omnivorous diet, not consuming DHA supplements during the last trimester, non-smoking, normal fetal Doppler scan and undergoing elective caesarean section. Subjects reporting health problems or pregnancy complications were excluded. Written informed consent was obtained from all subjects. This study was approved by the Hospital Ethics Committee.

### Sampling

Blood samples of fasted mothers were collected with EDTA-containing tubes at the time of delivery. Samples were centrifuged for 3 min at 1200 g to obtain plasma.

### Biochemical analyses

Insulin was analyzed by chemiluminescence (DIAsource INSIRMA, Nivelles, Belgium) and glucose, total cholesterol, TG, LDL cholesterol and HDL cholesterol were quantified by an automatic analyzer (Roche-Hitachi Modular PyD Autoanalyzer, Mannheim, Germany).

### Lipoprotein isolation

Maternal plasma lipoproteins were isolated from 1.5 mL of fresh plasma by ultracentrifugation using a discontinuous NaCl/KBr density-gradient [21] in an Optima L-100 XP ultracentifuge equipped with 100Ti rotor (Beckman Coulter, CA, USA). The rest of plasma was frozen in liquid nitrogen and stored at − 80 °C until analysis.

### Metabolomics measurement of the phospholipids

Metabolomics analysis was performed at the Division of Metabolic and Nutritional Medicine of the Dr. von Hauner Children’s Hospital in Munich, Germany, by liquid chromatography coupled to tandem mass spectrometry (LC-MS/MS), as described previously [12].

The analysis of polar lipids comprised diacyl-phosphatidylcholines (PCaa), acyl-alkyl-phosphatidylcholines (PCae), and SM. The analytical technique is not capable of determining the position of the double bonds and the distribution of carbon atoms between fatty acid side chains. The lipid species are described using the nomenclature CX:Y, where X is the length of the carbon chain (C), Y is the number of double bonds. “a” means, that the acyl chain is bound via an ester bond to the backbone and “e” means an ether bond.

For all metabolomics analyses, data acquisition on the mass spectrometer, data handling and quantification were performed with Analyst 1.6.2 software (AB Sciex, Darmstadt, Germany).

### Quality control

The quality control (QC) for the results was performed by using six QC samples per batch. Overall, two batches were measured with ten samples per lipoprotein species and plasma.

The QC was based on the inter- and intra-batch coefficient of variance (CV). For the intra batch CV, we used 20% and for the inter CV we used 30% as threshold. We excluded metabolites if more than 1 value of the QC samples exceeded 1.5 times the IQR. This was done, because with 6 QC samples, 2 values exceeding 1.5 times the IQR can be by chance.

### Statistics

The software R (3.0.2) (R Project for Statistical Computing, http://www.r-project.org/) was used for all statistical analyses in this study.

We graphically screened data for outlier and normal distribution. Critical values were defined as values that were 1.5 times the IQR above or below the median. Amongst the critical values, measurements that were two standard deviations apart from the next value were declared as influential observations.

Missing values were imputed using k nearest neighbour (knn) imputation [22], as a complete dataset is necessary for the calculation of percentages. If there were still influential observations left according to the above definition after knn imputation, the respective sample was excluded. We calculated the metabolite percentages for each individual and each species, to describe the relative phospholipid composition across lipoproteins. Percentages were calculated based on all metabolites and also metabolite group-wise (PCaa, SM, PCae). In a first step, we investigated the differences in phospholipid composition between lean and obese pregnant women, stratified by lipoprotein species. Wilcoxon Mann Whitney U test was applied, as with a small sample size (< 30), normal distribution cannot simply be assumed, and the Quantile-Quantile-plots indicated that the data was not normal distributed. The results were plotted in so called Manhattan plots (detailed results: Additional file 1: Tables S1-S6). In this plot, the |–log_10_(p)|-value is plotted on the y-axis and the metabolites on the x-axis. Values above the zero line show higher values of the metabolite in normal weight than in obese, values below show the opposite.

We also plotted metabolite concentrations against BMI as a continuous variable to see if there were tendencies in metabolite concentrations in dependency of the BMI (Additional file 1: Figures S1-S5).

In a second step, and as main aim of this analysis, we examined if the metabolite composition depended on the lipoprotein species. We conducted a principal component analysis (PCA) and produced score plots to investigate whether there was a grouping according to lipoprotein species.

In order to make results comparable for metabolites with low and high percentage levels, we calculated median ratios for each metabolite: For each pair (HDL/LDL, HDL/VLDL, LDL/VLDL), we calculated the medians in each species and built the ratio of these two. To obtain confidence intervals for these median ratios, we used 500 bootstrapping replicates. For each pairwise combination of lipoprotein species, we plotted the ratios together with their confidence interval, to depict the associations of the metabolites with the lipoproteins and to provide an estimate of the percent difference. Wilcoxon Mann Whitney U test was applied to see if there were significant differences in the phospholipid composition between the lipoprotein species (detailed results: Additional file 1: Tables S1-S6).

It was also analysed if the sum of SM to sum of PC ratio is significantly different between the lipoproteins.

The last step was to analyse the association between the significant phospholipids in the lipoprotein species with the cholesterol content of the respective lipoprotein to test for potential functional alignments of cholesterol and phospholipid species as hypothesised in previous studies [23]. This was done by Spearman correlation coefficients.

To account for multiple testing for the *p*-values, Bonferroni correction was used, which is done by dividing the p-level of 0.05 by the number of metabolites $\left(\frac{0.05}{71}=\kern0.5em 0.0007\right)$. Whenever *p*-values are reported, they are Bonferroni corrected. We define tendencies as uncorrected *p*-values of < 0.05.

## Results

**Fig. 1:**
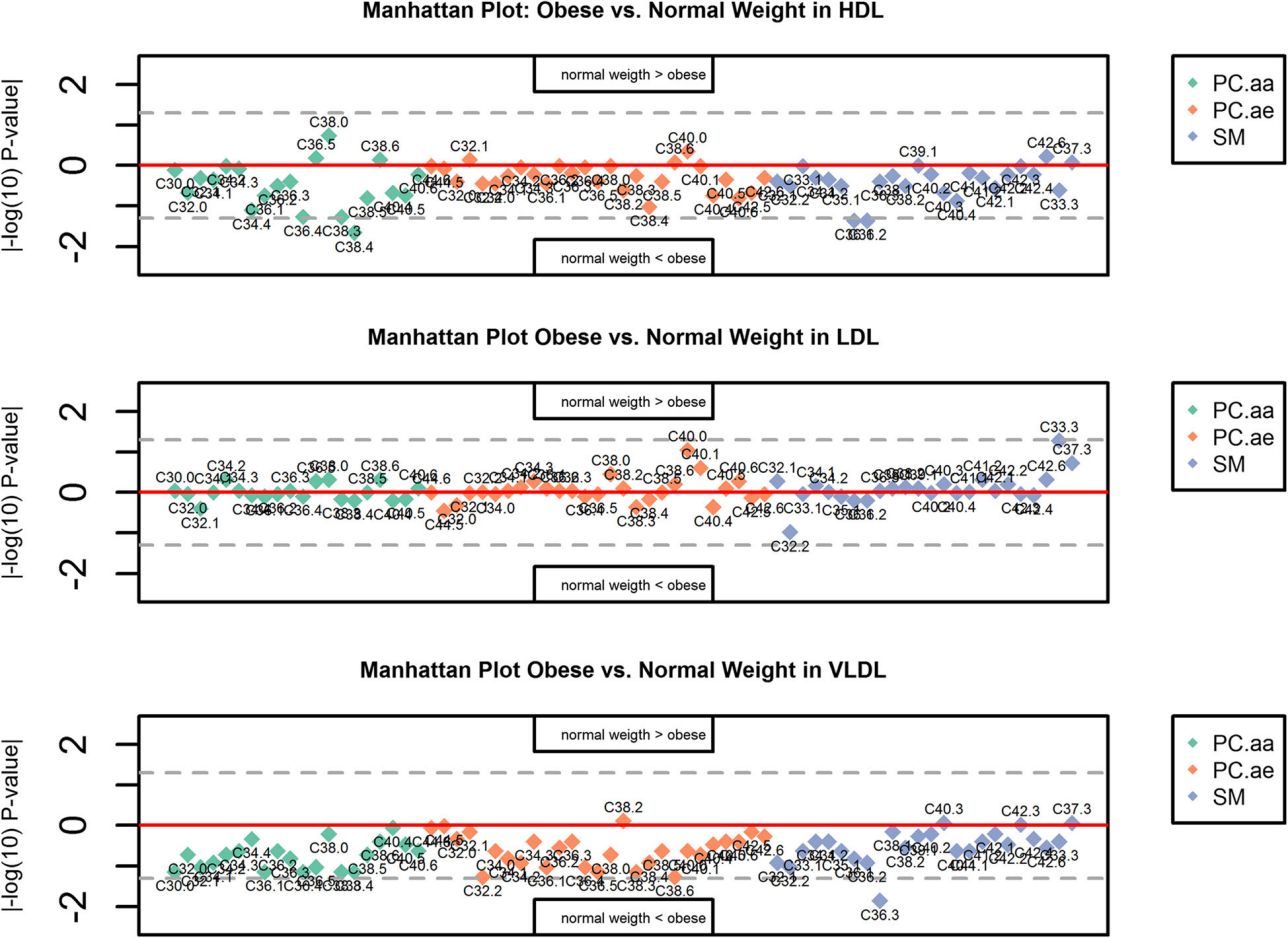
Manhattan plot showing the |–log_10_(p)| -values (y-axis) of the Wilcoxon Mann Whitney U test, testing for metabolite differences in plasma between normal weight and obese pregnant women, stratified by lipoprotein species. Scattered line: uncorrected α-level at ±log_10_(0.05) = ±1.3

Subject characteristics are shown in Table 1. A total of 71 PC and SM passed our QC criteria, comprising 20 PCaa (11 poly-unsaturated species), 27 SM (16 saturated and mono-unsaturated), and 24 PCae (15 polyunsaturated). Wilcoxon Mann Whitney U test for differences in metabolite concentrations between normal weight and obese subjects showed no significant differences after Bonferroni correction in any of the lipoprotein subsets, while a trend (uncorrected *p*-value < 0.05) towards a positive association with obesity was found for PCaa C38:4, SM C36:1 and SM C36:2 in HDL, and SM C36:3, PCae C32:2 and PCae C38:6 in VLDL (Fig. 1).

**Table 1:** Characteristics of pregnant women

	Control (*n* = 10)	Obese (*n* = 10)	*P*
Age (years)	33.80 ± 1.87	34.50 ± 2.31	0.807
Pregestational BMI (kg/m2)	22.51 ± 0.50	32.22 ± 0.92	< 0.001
BMI at delivery (kg/m2)	28.22 ± 0.57	35.63 ± 1.18	< 0.001
Glucose (mg/dl)	59.80 ± 2.48	62.10 ± 6.271	0.377
Insulin (UI)	9.47 ± 1.50	14.73 ± 2.68	0.090
TG (mmol/L)	1.94 ± 0.25	1.95 ± 0.17	0.973
Total cholesterol (mmol/L)	6.77 ± 0.40	6.31 ± 0.39	0.399
LDL cholesterol (mmol/L)	3.81 ± 0.31	3.37 ± 0.42	0.385
HDL cholesterol (mmol/L)	2.06 ± 0.24	2.00 ± 0.18	0.834

Results are expressed as mean ± SEM. Abbreviations: *BMI* body mass index, *TG* triacylglycerol, *HDL* high-density lipoprotein, *LDL* low-density lipoprotein

**Fig. 2:**
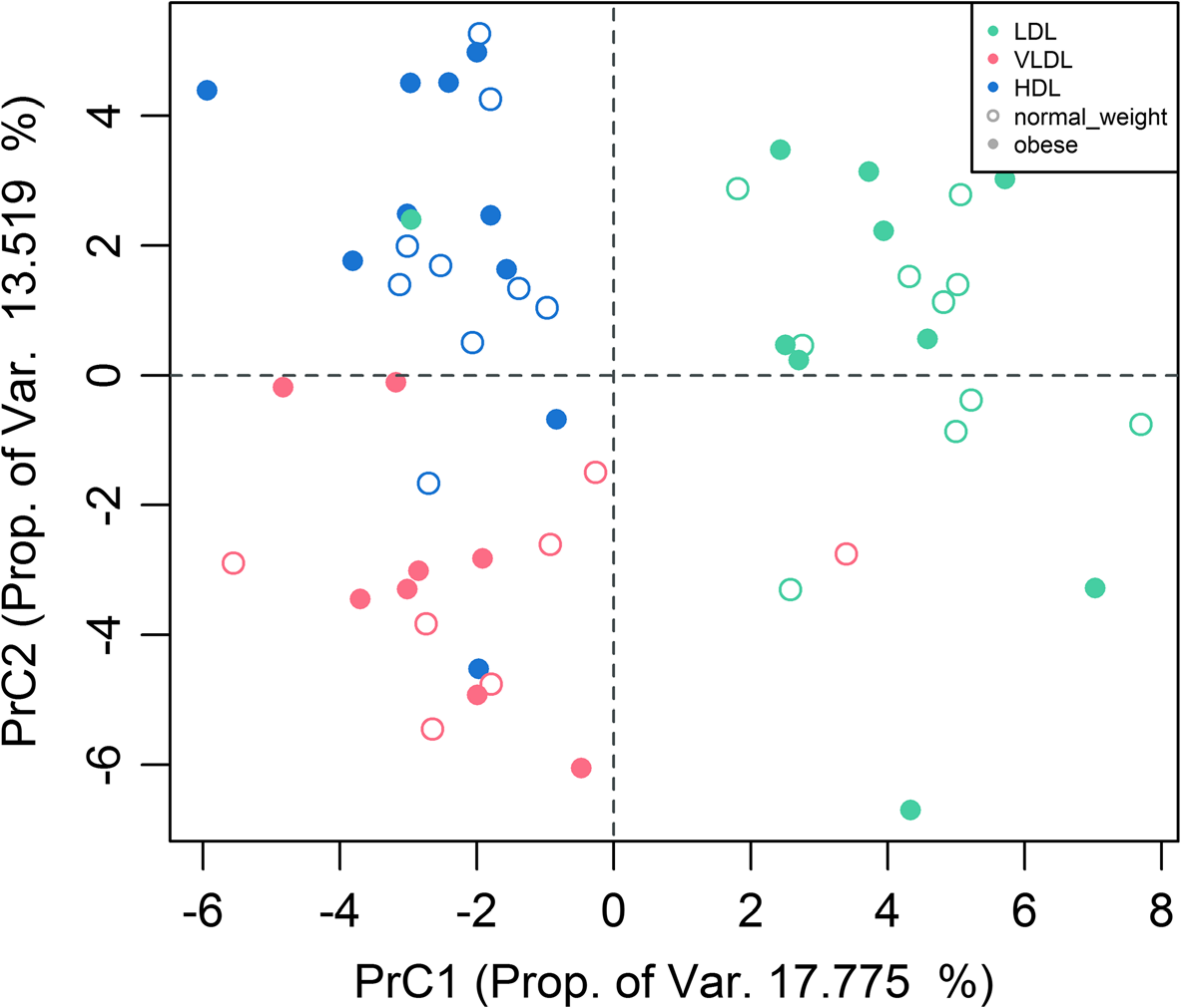
Score plot for the Principal Component Analysis of lipoprotein metabolite composition of normal weight and obese pregnant women

The PCA analysis showed a discriminatory trend between the lipoprotein species based on principal components (PrC) 1 and 2. The metabolites with highest scores in PrC 1 consisted mainly of SM species, and those in PrC2 of PC species (Fig. 2, Table 2). The score plot revealed that PrC1 mainly separates LDL from the other two, HDL and VLDL, whereas PrC2 separates VLDL from both LDL and HDL. The PCA confirms that phospholipid composition of obese and lean pregnant women is similar.

**Table 2:** Top 10 plasma lipoprotein Metabolites with highest absolute loadings in principal component 1 (PrC1) and 2 (PrC2)

Metabolite	PrC1	Metabolite	PrC2
SM C34:1	0.2633825	PCae C38:5	0.24530123
SM C36:1	0.23533697	PCae C38:4	0.24105136
SM C42:2	0.21961745	PCae C40:6	0.2195198
SM C41:1	0.215592	PCae C42:6	0.21406066
SM C34:2	0.20837699	PCaa C44:12	0.20189903
SM C42:3	0.20613141	PCae C36:3	0.19888559
PC C38:5	−0.20204572	PCaa C34:1	−0.19755011
SM C33:1	0.19581353	PCae C36:4	0.1966289
SM C32:1	0.1916571	PCaa C32:1	−0.19595342
SM C33:3	0.19136632	PCaa C34:2	−0.19438766

Wilcoxon Mann Whitney U test showed significant differences in the SM to PC ratios between the lipoproteins with a significantly higher ratio of SM/PC in LDL compared to HDL and VLDL (Fig. 4)

**Fig. 3:**
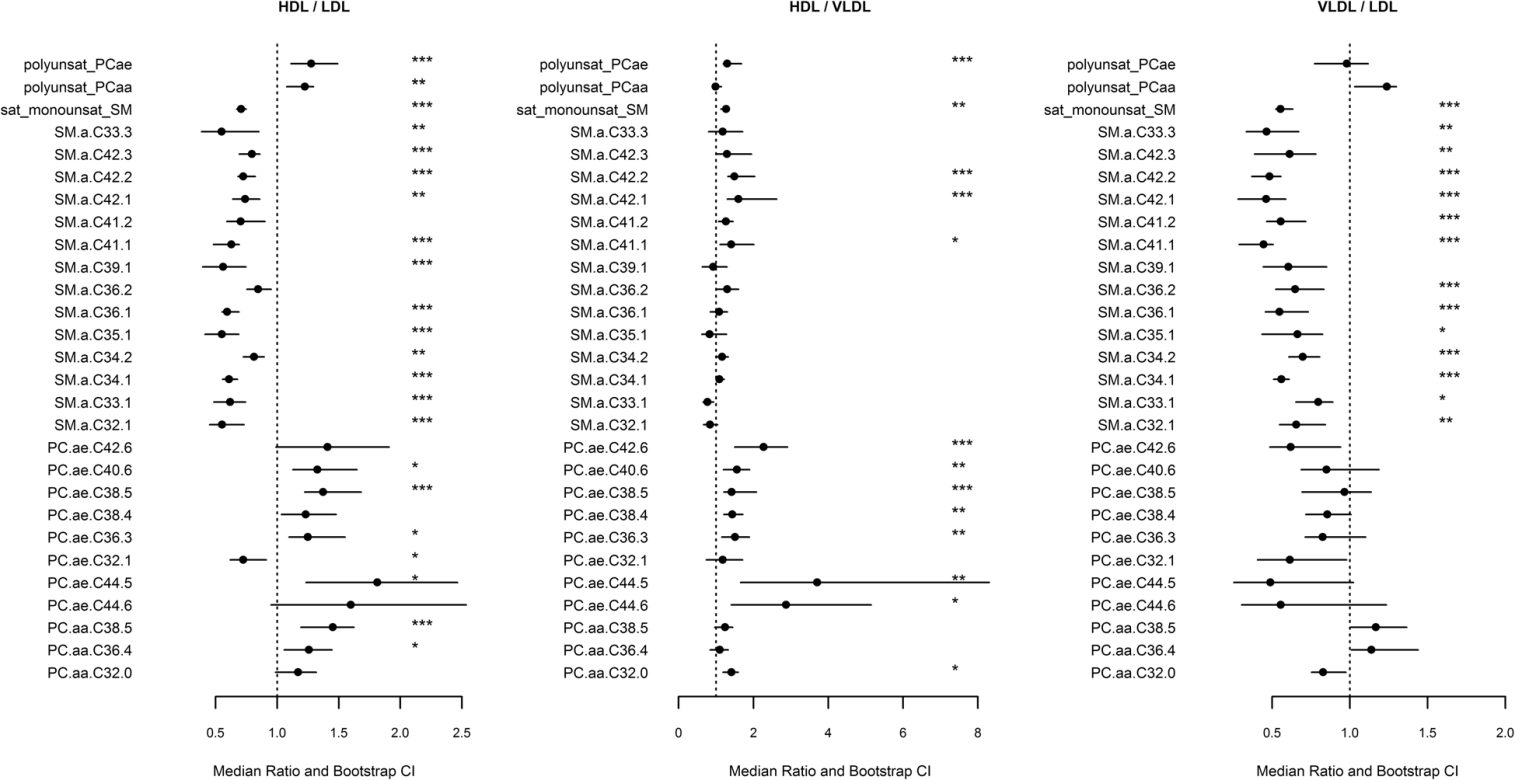
Plot of the ratios between the metabolite medians and sum of all mono-unsaturated sphingomyelins (SM) as well as poly-unsaturated ester-linked phosphatidylcholines (PCaa) and ether-linked phosphatidylcholines (PCae) in the different lipoprotein species. The 95% confidence intervals for the ratios are estimated by bootstrapping and significance level calculated by Wilcoxon Mann Whitney U-Test. The definition of the stars is the following: ‘*’ *P* < 0.05, ‘**‘*P* < 0.01, ‘***’ *P* < 0.001 . Only metabolites that are significantly different in at least one comparison are included. HDL, high density lipoprotein; LDL, low density lipoprotein; VLDL, very low density lipoprotein

When testing for differences between LDL and HDL in single metabolite species, LDL mainly contained saturated and monounsaturated SM. SM C36:1, SM C34:1, SM C32:1, SM C42:3, SM C41:1, SM C42:2, SM C35:1, SM C33:1, SM C39:1, SM C34:2, SM C33:3, SM C42:1 and PCae C32:1 were found significantly higher in LDL. Percentages of polyunsaturated fatty acids (PUFA) containing PC, PCaa C38:5, PCae C38:5, PCae C36:3, PCae C40:6, PCae C44:5 and PCaa C36:4 were significantly higher in HDL than in LDL (Fig. 3).

In the comparison between metabolites of the VLDL and HDL species, SM C42:2, PCae C38:5, SM C42:1, PCae C42:6, PCae C44:5, PCae C38:4, PCae C40:6, PCae C36:3, PCaa C32:0, PCae C44:6 and SM C41:1 have been significantly higher in HDL compared to VLDL. No metabolite was significantly higher in VLDL than in HDL (Fig. 3).

LDL showed significantly higher percentages of SM C42:2, SM C36:1, SM C41:1, SM C34:1, SM C42:1, SM C34:2, SM C41:2, SM C36:2, SM C33:3, SM C42:3, SM C32:1, SM C33:1 and SM C35:1 compared to VLDL (Fig. 3). In total, 25 (35%) of all measured phospholipids showed significant differences between lipoprotein species.

The Spearman correlation between the individual phospholipid species and total HDL-cholesterol and total LDL-cholesterol showed associations with different species, depending on the lipoprotein, but the scatterplots showed no clear tendencies (Additional file 1: Figures S1-S5). The top metabolites and cholesterol associations in HDL, with a correlation coefficient > 0.3 were PCae C32:1, PCae C36:3, PCae C38:5, PCaa C36:4, SM C33:3, SM C34:2, SM C34:1 and SM C32:1. In LDL the top associations between cholesterol and phospholipids were PCae C44:5, PCaa C38:5, SM C42:3, SM C42:1 and SM C32:1 (Additional file 1: Figures S1-S5).

## Discussion

The phospholipid composition of lipoproteins from 10 lean and 10 obese, pregnant women was not significantly different between the two groups.

Interestingly, the phospholipid species composition in the main lipoprotein species, LDL, HDL, and VLDL are similar to data reported previously for men and non-pregnant women [17] (Table 3).

**Table 3:** Comparison of our findings of associations between single phospholipid species percentages and lipoproteins with those of Wiesner et al. 2009 (Bold font: same direction, normal font: different direction)

Species	Wiesner et al. 2009	Our analysis
polyunsaturated	**HDL > LDL**	**HDL > LDL**
saturated/monounsaturated	**LDL > HDL**	**LDL > HDL**
PCaa C32:0	VLDL<LDL	VLDL>LDL
	**VLDL>HDL**	**VLDL>HDL**
	**LDL > HDL**	**LDL > HDL**
PCaa C36:4	**VLDL>LDL**	**VLDL>LDL**
	**VLDL<HDL**	**VLDL<HDL**
	**LDL < HDL**	**LDL < HDL**
PCaa C38:5	**VLDL>LDL**	**VLDL>LDL**
	**VLDL<HDL**	**VLDL<HDL**
	**LDL < HDL**	**LDL < HDL**
SM C32:1	VLDL>LDL	VLDL<LDL
	**VLDL>HDL**	**VLDL>HDL**
	**LDL > HDL**	**LDL > HDL**
SM C33:1	VLDL>LDL	VLDL<LDL
	**VLDL>HDL**	**VLDL>HDL**
	**LDL > HDL**	**LDL > HDL**
SM C34:2	VLDL>LDL	VLDL<LDL
	**VLDL<HDL**	**VLDL<HDL**
	LDL < HDL	LDL > LDL
SM C34:1	VLDL>LDL	VLDL<LDL
	VLDL>HDL	VLDL<HDL
	**LDL > HDL**	**LDL > HDL**
SM C35:1	VLDL>LDL	VLDL<LDL
	**VLDL>HDL**	**VLDL>HDL**
	**LDL > HDL**	**LDL > HDL**
SM C36:2	VLDL>LDL	VLDL<LDL
	**VLDL<HDL**	**VLDL<HDL**
	LDL < HDL	LDL > HDL
SM C36:1	**VLDL<LDL**	**VLDL<LDL**
	VLDL>HDL	VLDL<HDL
	**LDL > HDL**	**LDL > HDL**
SM C41:2	**VLDL<LDL**	**VLDL<LDL**
	**VLDL<HDL**	**VLDL<HDL**
	LDL < HDL	LDL > HDL
SM C41:1	**VLDL<LDL**	**VLDL<LDL**
	**VLDL<HDL**	**VLDL<HDL**
	**LDL > HDL**	**LDL > HDL**
SM C42:3	VLDL>LDL	VLDL<LDL
	**VLDL<HDL**	**VLDL<HDL**
	LDL < HDL	LDL > HDL
SM C42:2	**VLDL<LDL**	**VLDL<LDL**
	**VLDL<HDL**	**VLDL<HDL**
	LDL < HDL	LDL > HDL
SM C42:1	**VLDL<LDL**	**VLDL<LDL**
	**VLDL<HDL**	**VLDL<HDL**
	**LDL > HDL**	**LDL > HDL**
PCae C36:3	**VLDL<LDL**	**VLDL<LDL**
	**VLDL<HDL**	**VLDL <HDL**
	**LDL < HDL**	**LDL < HDL**
PCae C38:5	**VLDL<LDL**	**VLDL<LDL**
	**VLDL<HDL**	**VLDL<HDL**
	**LDL < HDL**	**LDL < HDL**
PCae C38:4	VLDL>LDL	VLDL<LDL
	**VLDL<HDL**	**VLDL<HDL**
	**LDL < HDL**	**LDL < HDL**
PCae C40:6	VLDL>LDL	VLDL<LDL
	VLDL>HDL	VLDL<HDL
	**LDL < HDL**	**LDL < HDL**

**Fig. 4:**
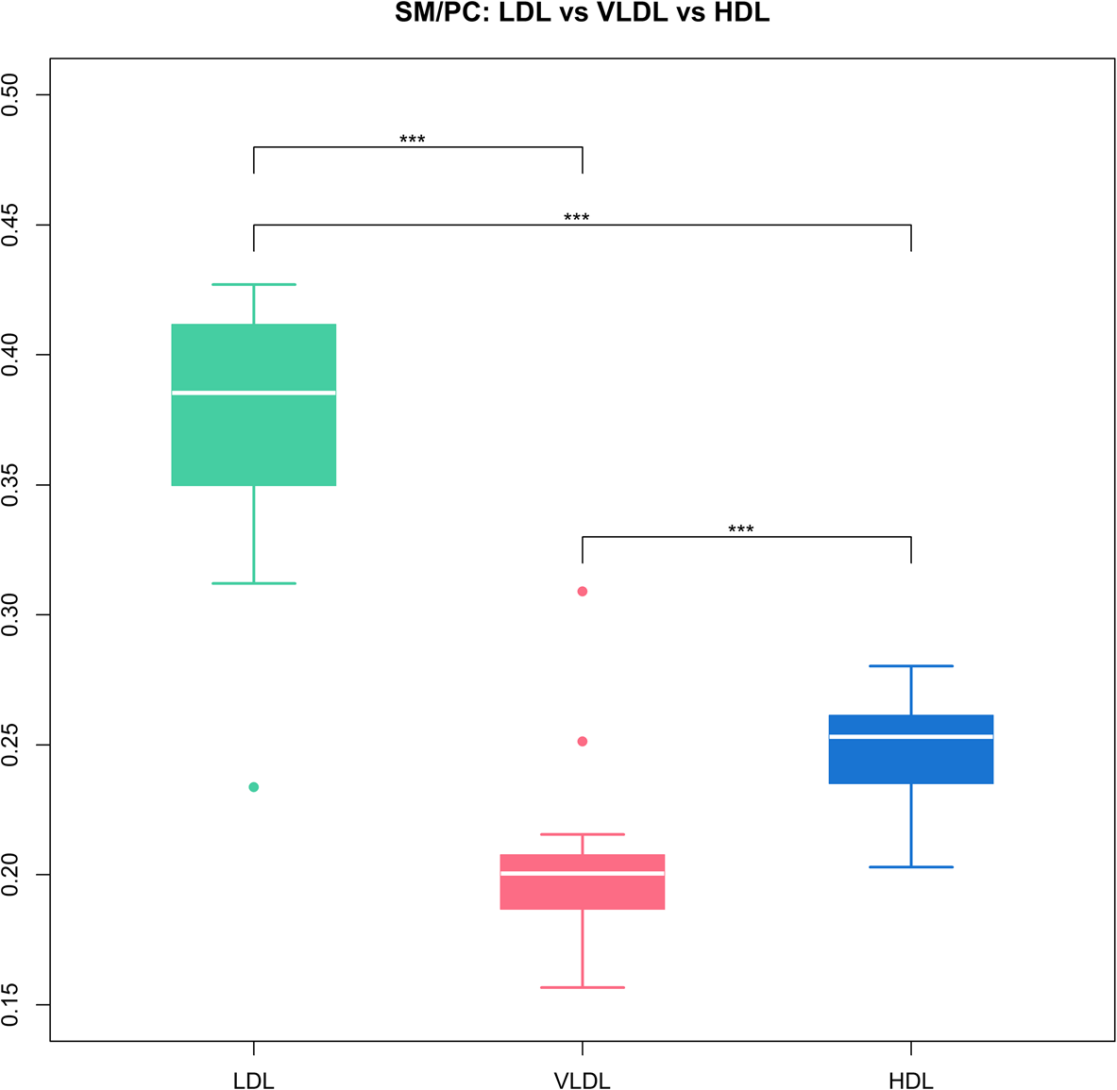
Boxplot of the sphingomyelins (SM) to phosphatidylcholines (PC) ratios in LDL, VLDL and HDL. Significance based on Wilcoxon Mann Whitney U Test. ***: *p*-value < 0.001. HDL, high density lipoprotein; LDL, low density lipoprotein; VLDL, very low density lipoprotein

We also found SM higher in LDL than VLDL or HDL, whereas HDL showed higher percentages of PC, predominantly PCae, than the other two lipoproteins. Metabolites associated with LDL contain more saturated and mono-unsaturated species, whilst the phospholipids found in higher proportions in HDL contained more polyunsaturated species. SM, in general, mainly consist of saturated and monounsaturated species [24]. LDL is associated with more saturated species and the established higher SM to PC ratio is indicative of that [25]. This study shows, that not only saturation or SM are associated more closely with LDL rather than HDL, but this is also SM species specific.

The phospholipid composition of the lipoproteins in the last trimester of the pregnancy is of particular importance, as this is when the placental lipid transfer mainly relies on the activity of the EL, which shows phospholipase rather than TG lipase activity [26]. Hence, especially the content of long-chain polyunsaturated and essential fatty acid species is important for the foetus. Those are mainly found in HDL [27], as confirmed in this study. It has been shown that SM has an inhibitory effect on EL, which is important especially for the LDL phospholipid uptake, although EL prefers HDL [28].

We show similar findings in pregnant women compared to male and non-pregnant women in other studies, that for example found LDL containing more SM C34:1 rather than VLDL or HDL [17, 29]. It appears quite possible that the association of lipoprotein patterns with cardiovascular and other diseases might actually be based on the composition of phospholipids in different lipoproteins.

Using absolute instead of relative values, previous research on associations of metabolite concentrations and waist circumference as a marker for obesity found that the metabolites SM C34:2, SM C36:2, SM C40:2, SM C42:3, PCaa C38:3, PCaa C40:5, PCaa C38:4, PCaa 40:6 and PCaa C38:5 were associated with obesity [12]. Our finding of a similar phospholipid pattern in the lipoproteins of normal weight and obese subjects might be due to the fact that higher lipids are part of a normal pregnancy in general and change rather dramatically during the course of gestation [30, 31], as confirmed by the lipoprotein and TAG levels in this study. Hence, hyperlipidemia as seen in obesity [32] might drive metabolic differences between normal weight and obese subjects, which did not emerge in this study, as both had higher lipid levels due to pregnancy. Therefore, it is important to consider that all women were pregnant, and especially have been in a situation in which plasma lipids increase gradually during gestation, reaching 2–3 times higher plasma fatty acids concentration at the end of pregnancy compared to non-pregnant women [33]. On the other hand, it is also possible that the small sample size of the lean vs. obese group in our study did not allow for detection of small group differences. Trends for differences were seen in two out of the six metabolites which have also been associated with obesity in a previous study (uncorrected *p*-value < 0.05) [12]. These are SM C36:2, and PCaa C38:4.

SM is known to negatively influence the activity of LCAT, as it is structurally similar to PC and is hence suggested to compete with PC for the receptors of the enzyme [34]. This might lead to more PC in the blood lipoproteins [35, 36].

The two main sites for SM production are either the liver or the intestine. The last and rate limiting step for the synthesis of SM is either sphingomyelin synthetase 1 or 2 (SMS1/SMS2) [37]. SMS1 is mainly located in the trans-Golgi, whereas SMS2 is located in the plasma membrane [37]. In the synthesis of sphingomyelin, the backbone can either be a sphingosine or another sphingoid base, mainly sphingadienine [17, 38].

It was proposed that lipoprotein SM might interact with cholesterol. The van der Waals forces between SM and cholesterol are strong, and it has been observed that not only in lipoproteins but also on the surface of any other cell, mainly consisting of phospholipids, the SM and cholesterol molecules are aligned [39]. This can also be explained by the saturated fatty acid side chain of SM and therefore a preferred binding [25]. The same is also apparent in lipid rafts [40], indicating a functional purpose of this assembly. This alignment was not apparent for SM/PC ratio or specific species in our study.

PCae are phospholipids that are characterized by an ether bond, usually localized at the sn-1 position of the glycerol-backbone (plasmalogens) [41]. PCae have previously been shown to be associated with apoA-I, which is the major protein in HDL [42]. Our finding of higher PCae values in HDL in comparison with the other lipoprotein species supports that. Also, it has been shown that PCae are needed to enable cholesterol efflux to HDL [42].

There are some studies analysing the functional contributions of phospholipids to the lipoproteins. For example, SM was shown to inhibit the activity of LCAT, as discussed above [30]. PCae have been shown to associate with cholesterol efflux from peripheral cells for the reverse cholesterol transport [43]. In a model experiment, a higher amount of saturated PCaa in the monolayer seemed to decrease the ability of HDL to accept free cholesterol [43], whereas in our study no clear trend for cholesterol and phospholipid preference could be shown.

Another interesting finding is the significant difference in phospholipid species between VLDL and LDL. VLDL and its phospholipid monolayer are formed in the liver and are a precursor of LDL, so the composition should be very similar. The difference might be according to the phospholipid and cholesteryl ester transfer proteins (PTP/CETP), which promote the exchange of cholesterol and phospholipids between the different phospholipid species [44]. This indicates, that the phospholipid composition of different lipoproteins might have direct effects on the others.

### Insights into functional significance

The phospholipid composition of lipoproteins in the third trimester of pregnancy, as shown in this study, is of particular interest, as it reflects an important source of fatty acids provided to the foetus. This is, as mentioned above, because at this stage in pregnancy, the placental lipid transfer mainly relies on the activity of the EL, showing predominantly phospholipase activity [26]. To support this, another study in pregnant women found decreased long-chain fatty acid concentrations in the mothers plasma in later pregnancy, which, as the authors suggest, may reflect conversion to triacylglycerols and phospholipids for energy supply to the foetus [45].

The tendency of obese pregnant women for higher concentrations of SM C36:2 and PCaa C38:4 in this study, previously related to obesity [12], might represent the hyperlipidemic state associated with both pregnancy [46] and obesity [47]. The phospholipid composition is of particular interest, as research has shown that pre-pregnancy diet might be associated with the composition of phospholipids, especially PCs containing dihomo-γ-linolenic acid species (20:3n-6) [48]. Dihomo-γ-linolenic acid is related to insulin resistance and adiposity in children [49, 50] and its concentrations throughout pregnancy in maternal blood are related to higher BMI of the offspring at age 6–7 [51, 52]. Obese mothers are more likely to give birth to children that develop obesity later on, so hence the phospholipid composition of lipoproteins might hold valuable insights both into dietary fatty acid intake before pregnancy, as well as biomarker for the offspring’s risk of developing obesity, holding the potential to unravel this intricate association network.

Further insight into these long-term associations has the potential for dietary recommendations in personalised medicine. In order to explore this association network, future studies are required to measure both the phospholipid composition of lipoproteins in pregnant mothers, as well as the non-esterified fatty acid and phospholipid concentrations in the offspring.

Further, most metabolomics studies have quantified serum or plasma metabolites without separation of lipoprotein species, which may have overlooked functional implications of lipoprotein composition that might well be related to the etiology of disorders such as diabetes, obesity or cardiovascular disease.

### Limitations

The small sample size with only twenty pregnant women limits the power of the study. Obese women were normolipidemic with respect to pregnancy, and our findings cannot be directly extrapolated to more extreme hyperlipidemic pregnant or non-pregnant obese women. Plasma samples were taken during caesarean section, which may imply possible alterations in blood metabolites secondary due to surgery-related stress, even though the similarity of metabolite patterns with reported data for non-pregnant adults suggests, that no major changes would have been induced.

Also, since the approach is novel, our institutional review board did not want us to study a large number of pregnant women before demonstrating that the method is feasible.

The strength of this study is the separation of individual phospholipid species in lipoproteins from lean and obese pregnant women. This method is demanding and not easy to scale-up to larger sample sizes. Despite the small sample size, we found significant associations with metabolites and lipoprotein species, which still persisted after correction for multiple testing.

## Conclusions

We showed for the first time different distributions of phospholipids in plasma lipoproteins of pregnant women, resembling those of men and non-pregnant women. This is indicating that the functionality of the lipoproteins might be influenced by molecular species composition independently of pregnancy status. More studies are needed to better understand the individual fatty acid and phospholipid composition of different lipoproteins to understand their implications for lipoprotein metabolism, health and disease.

## Additional file

Additional file 1: **Table S1.** Results for the Wilcoxon Mann Whitney U test for the association between single metabolite and normal weight versus obese subjects in HDL. **Table S2.** Results for the Wilcoxon Mann Whitney U test for the association between single metabolite and normal weight versus obese subjects in LDL. **Table S3.** Results for the Wilcoxon Mann Whitney U test for the association between single metabolite and normal weight versus obese subjects in VLDL. **Figure S1.** Scatterplots of Metabolite percentage and BMI for HDL. **Figure S2.** Scatterplots of Metabolite percentage and BMI for LDL. **Figure S3.** Scatterplots of Metabolite percentage and BMI for VLDL. **Table S4.** Results of the Wilcoxon Mann Whitney U Test for phospholipid differences between LDL and HDL. **Table S5.** Results of the Wilcoxon Mann Whitney U Test for phospholipid differences between VLDL and HDL. **Table S6.** Results of the Wilcoxon Mann Whitney U Test for phospholipid differences between VLDL and LDL. **Figure S4.** Scatterplot for the association of phospholipid species and cholesterol in HDL. **Figure S5.** Scatterplot for the association of phospholipid species and cholesterol in LDL. (DOCX 2509 kb)

## Abbreviations

ABCA1: ATP-binding cassette transporter A1
HDL: High density lipoprotein
IDL: Intermediate density lipoprotein
LCAT: Lecitin Colesterol Acil Transferasa
LDL: Low density lipoprotein
Lyso-PC: Lyso-phosphatidylcholine
PC: Phosphatidylcholine
PCA: Principal components analysis
PCaa: diacyl-phosphatidylcholine
PCae: acyl-alkylphophatidylcholine
PUFA: Polyunsaturated fatty acids
SM: Sphingomyelin
SMa: acyl-sphingomyelin
TG: Triacylglycerols
VLDL: Very low density lipoprotein
