# Supplementary material for: Phospholipids in lipoproteins: compositional differences across VLDL, LDL, and HDL in pregnant women
Source: Lipids Health Dis. 2019 Jan 22;18:20. doi: 10.1186/s12944-019-0957-z (PMC6343318; doi:10.1186/s12944-019-0957-z)
Supplement: Supplementary file 1 — Table S1. Results for the Wilcoxon Mann Whitney U test for the association between single metabolite and normal weight versus obese subjects in HDL. Table S2. Results for the Wilcoxon Mann Whitney U test for the association between single metabolite and normal weight versus obese subjects in LDL. Table S3. Results for the Wilcoxon Mann Whitney U test for the association between single metabolite and normal weight versus obese subjects in VLDL. Figure S1. Scatterplots of Metabolite percentage and BMI for HDL. Figure S2. Scatterplots of Metabolite percentage and BMI for LDL. Figure S3. Scatterplots of Metabolite percentage and BMI for VLDL. Table S4. Results of the Wilcoxon Mann Whitney U Test for phospholipid differences between LDL and HDL. Table S5. Results of the Wilcoxon Mann Whitney U Test for phospholipid differences between VLDL and HDL. Table S6. Results of the Wilcoxon Mann Whitney U Test for phospholipid differences between VLDL and LDL. Figure S4. Scatterplot for the association of phospholipid species and cholesterol in HDL. Figure S5. Scatterplot for the association of phospholipid species and cholesterol in LDL. (DOCX 2509 kb) [file 12944_2019_957_MOESM1_ESM.docx]

**Supplemental Material**

**Phospholipids in Lipoproteins: Compositional Differences Across VLDL, LDL, and HDL in Pregnant Women**

Sebastian Rauschert^1^, Antonio Gázquez^1-2^, Olaf Uhl^1^, Franca F. Kirchberg^1^, Hans Demmelmair^1^, María Ruíz-Palacios^2^, María T. Prieto-Sánchez^3^, José E. Blanco-Carnero^3^, Anibal Nieto^3^, Elvira Larqué^2^ and Berthold Koletzko^1^

1 Ludwig-Maximilians-Universität of Munich, Div. Metabolic and Nutritional Medicine, Dr. von Hauner Children´s Hospital, University of Munich Medical Center, Munich, 80337 Germany

2 Department of Physiology, Faculty of Biology, University of Murcia, Murcia, Spain

3 Obstetrics and Gynecology Service, Virgen de la Arrixaca Clinical Hospital, University of Murcia, Murcia, Spain

**Corresponding author:**

Berthold Koletzko, MD PhD (Dr med Dr med habil), Professor of Pediatrics

Dr. von Hauner Children’s Hospital

Ludwig-Maximilians-Universität Munich

Lindwurmstr. 4, D-80337 München, Germany

Phone: +49 89 44005 2826

Fax: +49 89 44005 7742

E-Mail: [office.koletzko@med.uni-muenchen.de](mailto:office.koletzko@med.uni-muenchen.de)

**Content**

[**Supplement 1.1:** Results for the Wilcoxon Mann Whitney U test for the association between single metabolite and normal weight versus obese subjects in HDL. 3](#_Toc491088137)

[**Supplement 1.2:** Results for the Wilcoxon Mann Whitney U test for the association between single metabolite and normal weight versus obese subjects in LDL. 6](#_Toc491088138)

[**Supplement 1.3:** Results for the Wilcoxon Mann Whitney U test for the association between single metabolite and normal weight versus obese subjects in VLDL. 9](#_Toc491088139)

[**Supplement 2.1:** Scatterplots of Metabolite percentage and BMI for HDL 12](#_Toc491088140)

[**Supplement 2.2:** Scatterplots of Metabolite percentage and BMI for LDL 20](#_Toc491088141)

[**Supplement 2.3:** Scatterplots of Metabolite percentage and BMI for VLDL 28](#_Toc491088142)

[**Supplement 3.1:** Results of the Wilcoxon Mann Whitney U Test for phospholipid differences between LDL and HDL 36](#_Toc491088143)

[**Supplement 3.2:** Results of the Wilcoxon Mann Whitney U Test for phospholipid differences between VLDL and HDL 38](#_Toc491088144)

[**Supplement 3.2:** Results of the Wilcoxon Mann Whitney U Test for phospholipid differences between VLDL and LDL 40](#_Toc491088145)

[**Supplement 4.1:** Scatterplot for the association of phospholipid species and cholesterol in HDL 42](#_Toc491088146)

[**Supplement 4.2:** Scatterplot for the association of phospholipid species and cholesterol in LDL 47](#_Toc491088147)

# Supplement 1.1: Results for the Wilcoxon Mann Whitney U test for the association between single metabolite and normal weight versus obese subjects in HDL.

#

| **Analytes** | **P-Value** | **Bonferroni P-Value** | **Mean Obese** | **Mean Normal** | **Mean obese - Mean Normal** |
| --- | --- | --- | --- | --- | --- |
| PC.aa.C30.0 | 0.78018576 | 1 | 0.141916976 | 0.194019647 | -0.052102671 |
| PC.aa.C32.0 | 0.21102427 | 1 | 0.559641931 | 0.834120776 | -0.274478846 |
| PC.aa.C32.1 | 0.49669835 | 1 | 0.724585003 | 1.134078131 | -0.409493128 |
| PC.aa.C34.1 | 0.44696789 | 1 | 12.38642658 | 17.52271347 | -5.136286897 |
| PC.aa.C34.2 | 0.96823919 | 1 | 27.10106225 | 35.79033492 | -8.68927267 |
| PC.aa.C34.3 | 0.84210526 | 1 | 0.53888627 | 0.65835482 | -0.119468551 |
| PC.aa.C34.4 | 0.07889324 | 1 | 0.06622894 | 0.08197325 | -0.01574431 |
| PC.aa.C36.1 | 0.18231614 | 1 | 1.786698993 | 2.587659284 | -0.800960291 |
| PC.aa.C36.2 | 0.31537812 | 1 | 9.51927262 | 13.67255471 | -4.153282091 |
| PC.aa.C36.3 | 0.40018186 | 1 | 6.93164141 | 10.54355022 | -3.611908808 |
| PC.aa.C36.4 | 0.05347594 | 1 | 9.097853897 | 16.42719167 | -7.329337772 |
| PC.aa.C36.5 | 0.66072008 | 1 | 0.337755326 | 0.463744578 | -0.125989251 |
| PC.aa.C38.0 | 0.18231614 | 1 | 0.159710488 | 0.227480323 | -0.067769835 |
| PC.aa.C38.3 | 0.05347594 | 1 | 1.718076206 | 3.172652823 | -1.454576618 |
| PC.aa.C38.4 | 0.02201823 | 1 | 3.806873324 | 7.022899729 | -3.216026405 |
| PC.aa.C38.5 | 0.15640087 | 1 | 1.5783509 | 2.472580492 | -0.894229592 |
| PC.aa.C38.6 | 0.71969517 | 1 | 5.443913562 | 6.638423638 | -1.194510076 |
| PC.aa.C40.4 | 0.21102427 | 1 | 0.165106875 | 0.200372972 | -0.035266097 |
| PC.aa.C40.5 | 0.18231614 | 1 | 0.421061301 | 0.556655394 | -0.135594093 |
| PC.aa.C40.6 | 0.60378012 | 1 | 1.290990329 | 1.797643214 | -0.506652885 |
| PC.ae.C44.6 | 0.96823919 | 1 | 0.104528986 | 0.145285467 | -0.040756481 |
| PC.ae.C44.5 | 0.84210526 | 1 | 0.022809399 | 0.021175641 | 0.001633757 |
| PC.ae.C32.0 | 0.40018186 | 1 | 0.105295056 | 0.124251931 | -0.018956875 |
| PC.ae.C32.1 | 0.71969517 | 1 | 0.100091027 | 0.107520375 | -0.007429349 |
| PC.ae.C32.2 | 0.356232 | 1 | 0.018139096 | 0.044938636 | -0.02679954 |
| PC.ae.C34.0 | 0.356232 | 1 | 0.030432025 | 0.098778257 | -0.068346232 |
| PC.ae.C34.1 | 0.54896187 | 1 | 0.380908288 | 0.503361779 | -0.122453491 |
| PC.ae.C34.2 | 0.90482582 | 1 | 0.395833222 | 0.547993897 | -0.152160675 |
| PC.ae.C34.3 | 0.60378012 | 1 | 0.244064819 | 0.318721555 | -0.074656736 |
| PC.ae.C36.1 | 0.356232 | 1 | 0.255745337 | 0.365909878 | -0.11016454 |
| PC.ae.C36.2 | 0.96823919 | 1 | 0.595903772 | 0.995468778 | -0.399565006 |
| PC.ae.C36.3 | 0.66072008 | 1 | 0.235607864 | 0.343345083 | -0.107737219 |
| PC.ae.C36.4 | 0.90482582 | 1 | 0.464080939 | 0.867153712 | -0.403072772 |
| PC.ae.C36.5 | 0.40018186 | 1 | 0.314086569 | 0.67711176 | -0.363025191 |
| PC.ae.C38.0 | 0.96823919 | 1 | 0.060792938 | 0.092042184 | -0.031249245 |
| PC.ae.C38.2 | 0.24280673 | 1 | 0.11151287 | 0.105767199 | 0.005745671 |
| PC.ae.C38.3 | 0.54896187 | 1 | 0.104771957 | 0.172109378 | -0.067337421 |
| PC.ae.C38.4 | 0.09471952 | 1 | 0.361712908 | 0.693108165 | -0.331395257 |
| PC.ae.C38.5 | 0.40018186 | 1 | 0.568150015 | 0.853480671 | -0.285330656 |
| PC.ae.C38.6 | 0.84210526 | 1 | 0.22704841 | 0.417973531 | -0.190925121 |
| PC.ae.C40.0 | 0.44696789 | 1 | 0.699867326 | 0.899617342 | -0.199750016 |
| PC.ae.C40.1 | 0.96823919 | 1 | 0.063092945 | 0.089212077 | -0.026119132 |
| PC.ae.C40.4 | 0.18231614 | 1 | 0.110660247 | 0.139886189 | -0.029225943 |
| PC.ae.C40.5 | 0.44696789 | 1 | 0.140206054 | 0.153470111 | -0.013264057 |
| PC.ae.C40.6 | 0.15640087 | 1 | 0.14591329 | 0.205640465 | -0.059727175 |
| PC.ae.C42.5 | 0.21102427 | 1 | 0.11155795 | 0.113279327 | -0.001721378 |
| PC.ae.C42.6 | 0.49669835 | 1 | 0.055373779 | 0.089970631 | -0.034596852 |
| SM.a.C32.1 | 0.40018186 | 1 | 0.355152934 | 0.409967029 | -0.054814095 |
| SM.a.C32.2 | 0.31537812 | 1 | 0.035753696 | 0.053081342 | -0.017327646 |
| SM.a.C33.1 | 0.96823919 | 1 | 0.247579588 | 0.37696106 | -0.129381472 |
| SM.a.C34.1 | 0.49669835 | 1 | 5.179827814 | 6.268065147 | -1.088237333 |
| SM.a.C34.2 | 0.44696789 | 1 | 0.814313826 | 1.072587695 | -0.258273869 |
| SM.a.C35.1 | 0.31537812 | 1 | 0.152491966 | 0.215174547 | -0.062682581 |
| SM.a.C36.1 | 0.04347355 | 1 | 1.050398378 | 1.573288421 | -0.522890043 |
| SM.a.C36.2 | 0.04347355 | 1 | 0.420665573 | 0.69336259 | -0.272697016 |
| SM.a.C36.3 | 0.40018186 | 1 | 0.025439821 | 0.051804061 | -0.026364241 |
| SM.a.C38.1 | 0.54896187 | 1 | 1.948259355 | 1.870187223 | 0.078072131 |
| SM.a.C38.2 | 0.31537812 | 1 | 1.012066201 | 1.306872669 | -0.294806468 |
| SM.a.C39.1 | 1 | 1 | 0.277887826 | 0.322361221 | -0.044473395 |
| SM.a.C40.2 | 0.60378012 | 1 | 1.600380905 | 1.731704537 | -0.131323632 |
| SM.a.C40.3 | 0.21102427 | 1 | 0.437720863 | 0.389810533 | 0.047910329 |
| SM.a.C40.4 | 0.13330014 | 1 | 0.003778836 | 0.005962717 | -0.002183881 |
| SM.a.C41.1 | 0.66072008 | 1 | 0.596735143 | 0.798753784 | -0.202018641 |
| SM.a.C41.2 | 0.49669835 | 1 | 0.570236765 | 0.659490694 | -0.089253929 |
| SM.a.C42.1 | 0.27751196 | 1 | 0.963239108 | 1.263573697 | -0.300334589 |
| SM.a.C42.2 | 0.60378012 | 1 | 2.820234397 | 3.41634391 | -0.596109513 |
| SM.a.C42.3 | 0.96823919 | 1 | 1.684966909 | 1.587890795 | 0.097076114 |
| SM.a.C42.4 | 0.60378012 | 1 | 0.38610567 | 0.535047023 | -0.148941353 |
| SM.a.C42.6 | 0.60378012 | 1 | 0.160845719 | 0.231540395 | -0.070694676 |
| SM.a.C33.3 | 0.24280673 | 1 | 0.005710425 | 0.006430681 | -0.000720257 |
| SM.a.C37.3 | 0.84210526 | 1 | 0.014551445 | 0.012346044 | 0.0022054 |

# Supplement 1.2: Results for the Wilcoxon Mann Whitney U test for the association between single metabolite and normal weight versus obese subjects in LDL.

| **Analytes** | **P-Value** | **Bonferroni P-Value** | **Mean Obese** | **Mean Normal** | **Mean obese - Mean Normal** |
| --- | --- | --- | --- | --- | --- |
| PC.aa.C30.0 | 0.911797181 | 1 | 0.141916976 | 0.194019647 | -0.052102671 |
| PC.aa.C32.0 | 0.911797181 | 1 | 0.559641931 | 0.834120776 | -0.274478846 |
| PC.aa.C32.1 | 0.393048128 | 1 | 0.724585003 | 1.134078131 | -0.409493128 |
| PC.aa.C34.1 | 1 | 1 | 12.38642658 | 17.52271347 | -5.136286897 |
| PC.aa.C34.2 | 0.481250947 | 1 | 27.10106225 | 35.79033492 | -8.68927267 |
| PC.aa.C34.3 | 0.911797181 | 1 | 0.53888627 | 0.65835482 | -0.119468551 |
| PC.aa.C34.4 | 0.853428305 | 1 | 0.06622894 | 0.08197325 | -0.01574431 |
| PC.aa.C36.1 | 0.795936262 | 1 | 1.786698993 | 2.587659284 | -0.800960291 |
| PC.aa.C36.2 | 0.911797181 | 1 | 9.51927262 | 13.67255471 | -4.153282091 |
| PC.aa.C36.3 | 0.911797181 | 1 | 6.93164141 | 10.54355022 | -3.611908808 |
| PC.aa.C36.4 | 0.795936262 | 1 | 9.097853897 | 16.42719167 | -7.329337772 |
| PC.aa.C36.5 | 0.52884886 | 1 | 0.337755326 | 0.463744578 | -0.125989251 |
| PC.aa.C38.0 | 0.481250947 | 1 | 0.159710488 | 0.227480323 | -0.067769835 |
| PC.aa.C38.3 | 0.684210526 | 1 | 1.718076206 | 3.172652823 | -1.454576618 |
| PC.aa.C38.4 | 0.630528914 | 1 | 3.806873324 | 7.022899729 | -3.216026405 |
| PC.aa.C38.5 | 1 | 1 | 1.5783509 | 2.472580492 | -0.894229592 |
| PC.aa.C38.6 | 0.481250947 | 1 | 5.443913562 | 6.638423638 | -1.194510076 |
| PC.aa.C40.4 | 0.630528914 | 1 | 0.165106875 | 0.200372972 | -0.035266097 |
| PC.aa.C40.5 | 0.684210526 | 1 | 0.421061301 | 0.556655394 | -0.135594093 |
| PC.aa.C40.6 | 0.795936262 | 1 | 1.290990329 | 1.797643214 | -0.506652885 |
| PC.ae.C44.6 | 1 | 1 | 0.104528986 | 0.145285467 | -0.040756481 |
| PC.ae.C44.5 | 0.352681374 | 1 | 0.022809399 | 0.021175641 | 0.001633757 |
| PC.ae.C32.0 | 0.481250947 | 1 | 0.105295056 | 0.124251931 | -0.018956875 |
| PC.ae.C32.1 | 0.97051246 | 1 | 0.100091027 | 0.107520375 | -0.007429349 |
| PC.ae.C32.2 | 0.97051246 | 1 | 0.018139096 | 0.044938636 | -0.02679954 |
| PC.ae.C34.0 | 0.911797181 | 1 | 0.030432025 | 0.098778257 | -0.068346232 |
| PC.ae.C34.1 | 0.911797181 | 1 | 0.380908288 | 0.503361779 | -0.122453491 |
| PC.ae.C34.2 | 0.739364351 | 1 | 0.395833222 | 0.547993897 | -0.152160675 |
| PC.ae.C34.3 | 0.52884886 | 1 | 0.244064819 | 0.318721555 | -0.074656736 |
| PC.ae.C36.1 | 0.795936262 | 1 | 0.255745337 | 0.365909878 | -0.11016454 |
| PC.ae.C36.2 | 0.911797181 | 1 | 0.595903772 | 0.995468778 | -0.399565006 |
| PC.ae.C36.3 | 0.911797181 | 1 | 0.235607864 | 0.343345083 | -0.107737219 |
| PC.ae.C36.4 | 0.795936262 | 1 | 0.464080939 | 0.867153712 | -0.403072772 |
| PC.ae.C36.5 | 0.911797181 | 1 | 0.314086569 | 0.67711176 | -0.363025191 |
| PC.ae.C38.0 | 0.352681374 | 1 | 0.060792938 | 0.092042184 | -0.031249245 |
| PC.ae.C38.2 | 0.795936262 | 1 | 0.11151287 | 0.105767199 | 0.005745671 |
| PC.ae.C38.3 | 0.435872177 | 1 | 0.104771957 | 0.172109378 | -0.067337421 |
| PC.ae.C38.4 | 0.684210526 | 1 | 0.361712908 | 0.693108165 | -0.331395257 |
| PC.ae.C38.5 | 1 | 1 | 0.568150015 | 0.853480671 | -0.285330656 |
| PC.ae.C38.6 | 0.630528914 | 1 | 0.22704841 | 0.417973531 | -0.190925121 |
| PC.ae.C40.0 | 0.089209552 | 1 | 0.699867326 | 0.899617342 | -0.199750016 |
| PC.ae.C40.1 | 0.247450692 | 1 | 0.063092945 | 0.089212077 | -0.026119132 |
| PC.ae.C40.4 | 0.435872177 | 1 | 0.110660247 | 0.139886189 | -0.029225943 |
| PC.ae.C40.5 | 0.795936262 | 1 | 0.140206054 | 0.153470111 | -0.013264057 |
| PC.ae.C40.6 | 0.52884886 | 1 | 0.14591329 | 0.205640465 | -0.059727175 |
| PC.ae.C42.5 | 0.739364351 | 1 | 0.11155795 | 0.113279327 | -0.001721378 |
| PC.ae.C42.6 | 0.911797181 | 1 | 0.055373779 | 0.089970631 | -0.034596852 |
| SM.a.C32.1 | 0.52884886 | 1 | 0.355152934 | 0.409967029 | -0.054814095 |
| SM.a.C32.2 | 0.105122432 | 1 | 0.035753696 | 0.053081342 | -0.017327646 |
| SM.a.C33.1 | 0.911797181 | 1 | 0.247579588 | 0.37696106 | -0.129381472 |
| SM.a.C34.1 | 0.684210526 | 1 | 5.179827814 | 6.268065147 | -1.088237333 |
| SM.a.C34.2 | 0.97051246 | 1 | 0.814313826 | 1.072587695 | -0.258273869 |
| SM.a.C35.1 | 0.795936262 | 1 | 0.152491966 | 0.215174547 | -0.062682581 |
| SM.a.C36.1 | 0.630528914 | 1 | 1.050398378 | 1.573288421 | -0.522890043 |
| SM.a.C36.2 | 0.630528914 | 1 | 0.420665573 | 0.69336259 | -0.272697016 |
| SM.a.C36.3 | 0.911797181 | 1 | 0.025439821 | 0.051804061 | -0.026364241 |
| SM.a.C38.1 | 0.795936262 | 1 | 1.948259355 | 1.870187223 | 0.078072131 |
| SM.a.C38.2 | 0.739364351 | 1 | 1.012066201 | 1.306872669 | -0.294806468 |
| SM.a.C39.1 | 0.795936262 | 1 | 0.277887826 | 0.322361221 | -0.044473395 |
| SM.a.C40.2 | 0.97051246 | 1 | 1.600380905 | 1.731704537 | -0.131323632 |
| SM.a.C40.3 | 0.630528914 | 1 | 0.437720863 | 0.389810533 | 0.047910329 |
| SM.a.C40.4 | 0.97051246 | 1 | 0.003778836 | 0.005962717 | -0.002183881 |
| SM.a.C41.1 | 0.97051246 | 1 | 0.596735143 | 0.798753784 | -0.202018641 |
| SM.a.C41.2 | 0.481250947 | 1 | 0.570236765 | 0.659490694 | -0.089253929 |
| SM.a.C42.1 | 0.911797181 | 1 | 0.963239108 | 1.263573697 | -0.300334589 |
| SM.a.C42.2 | 0.630528914 | 1 | 2.820234397 | 3.41634391 | -0.596109513 |
| SM.a.C42.3 | 0.911797181 | 1 | 1.684966909 | 1.587890795 | 0.097076114 |
| SM.a.C42.4 | 0.853428305 | 1 | 0.38610567 | 0.535047023 | -0.148941353 |
| SM.a.C42.6 | 0.481250947 | 1 | 0.160845719 | 0.231540395 | -0.070694676 |
| SM.a.C33.3 | 0.052425902 | 1 | 0.005710425 | 0.006430681 | -0.000720257 |
| SM.a.C37.3 | 0.190315876 | 1 | 0.014551445 | 0.012346044 | 0.0022054 |

# Supplement 1.3: Results for the Wilcoxon Mann Whitney U test for the association between single metabolite and normal weight versus obese subjects in VLDL.

| **Analytes** | **P-Value** | **Bonferroni P-Value** | **Mean Obese** | **Mean Normal** | **Mean obese - Mean Normal** |
| --- | --- | --- | --- | --- | --- |
| PC.aa.C30.0 | 0.07210567 | 1 | 0.14191698 | 0.19401965 | -0.05210267 |
| PC.aa.C32.0 | 0.18927739 | 1 | 0.55964193 | 0.83412078 | -0.27447885 |
| PC.aa.C32.1 | 0.09386169 | 1 | 0.724585 | 1.13407813 | -0.40949313 |
| PC.aa.C34.1 | 0.12059052 | 1 | 12.3864266 | 17.5227135 | -5.1362869 |
| PC.aa.C34.2 | 0.18927739 | 1 | 27.1010622 | 35.7903349 | -8.68927267 |
| PC.aa.C34.3 | 0.23185703 | 1 | 0.53888627 | 0.65835482 | -0.11946855 |
| PC.aa.C34.4 | 0.46340326 | 1 | 0.06622894 | 0.08197325 | -0.01574431 |
| PC.aa.C36.1 | 0.07210567 | 1 | 1.78669899 | 2.58765928 | -0.80096029 |
| PC.aa.C36.2 | 0.23185703 | 1 | 9.51927262 | 13.6725547 | -4.15328209 |
| PC.aa.C36.3 | 0.15198135 | 1 | 6.93164141 | 10.5435502 | -3.61190881 |
| PC.aa.C36.4 | 0.07210567 | 1 | 9.0978539 | 16.4271917 | -7.32933777 |
| PC.aa.C36.5 | 0.09386169 | 1 | 0.33775533 | 0.46374458 | -0.12598925 |
| PC.aa.C38.0 | 0.61258741 | 1 | 0.15971049 | 0.22748032 | -0.06776983 |
| PC.aa.C38.3 | 0.07210567 | 1 | 1.71807621 | 3.17265282 | -1.45457662 |
| PC.aa.C38.4 | 0.07210567 | 1 | 3.80687332 | 7.02289973 | -3.2160264 |
| PC.aa.C38.5 | 0.18927739 | 1 | 1.5783509 | 2.47258049 | -0.89422959 |
| PC.aa.C38.6 | 0.396892 | 1 | 5.44391356 | 6.63842364 | -1.19451008 |
| PC.aa.C40.4 | 0.86651127 | 1 | 0.16510687 | 0.20037297 | -0.0352661 |
| PC.aa.C40.5 | 0.33566434 | 1 | 0.4210613 | 0.55665539 | -0.13559409 |
| PC.aa.C40.6 | 0.23185703 | 1 | 1.29099033 | 1.79764321 | -0.50665289 |
| PC.ae.C44.6 | 0.86651127 | 1 | 0.10452899 | 0.14528547 | -0.04075648 |
| PC.ae.C44.5 | 0.95508936 | 1 | 0.0228094 | 0.02117564 | 0.00163376 |
| PC.ae.C32.0 | 0.46340326 | 1 | 0.10529506 | 0.12425193 | -0.01895687 |
| PC.ae.C32.1 | 0.69432789 | 1 | 0.10009103 | 0.10752038 | -0.00742935 |
| PC.ae.C32.2 | 0.05407925 | 1 | 0.0181391 | 0.04493864 | -0.02679954 |
| PC.ae.C34.0 | 0.23185703 | 1 | 0.03043202 | 0.09877826 | -0.06834623 |
| PC.ae.C34.1 | 0.15198135 | 1 | 0.38090829 | 0.50336178 | -0.12245349 |
| PC.ae.C34.2 | 0.12059052 | 1 | 0.39583322 | 0.5479939 | -0.15216067 |
| PC.ae.C34.3 | 0.396892 | 1 | 0.24406482 | 0.31872155 | -0.07465674 |
| PC.ae.C36.1 | 0.09386169 | 1 | 0.25574534 | 0.36590988 | -0.11016454 |
| PC.ae.C36.2 | 0.28096348 | 1 | 0.59590377 | 0.99546878 | -0.39956501 |
| PC.ae.C36.3 | 0.396892 | 1 | 0.23560786 | 0.34334508 | -0.10773722 |
| PC.ae.C36.4 | 0.09386169 | 1 | 0.46408094 | 0.86715371 | -0.40307277 |
| PC.ae.C36.5 | 0.07210567 | 1 | 0.31408657 | 0.67711176 | -0.36302519 |
| PC.ae.C38.0 | 0.18927739 | 1 | 0.06079294 | 0.09204218 | -0.03124925 |
| PC.ae.C38.2 | 0.77886558 | 1 | 0.11151287 | 0.1057672 | 0.00574567 |
| PC.ae.C38.3 | 0.07210567 | 1 | 0.10477196 | 0.17210938 | -0.06733742 |
| PC.ae.C38.4 | 0.12059052 | 1 | 0.36171291 | 0.69310817 | -0.33139526 |
| PC.ae.C38.5 | 0.23185703 | 1 | 0.56815001 | 0.85348067 | -0.28533066 |
| PC.ae.C38.6 | 0.05407925 | 1 | 0.22704841 | 0.41797353 | -0.19092512 |
| PC.ae.C40.0 | 0.23185703 | 1 | 0.69986733 | 0.89961734 | -0.19975002 |
| PC.ae.C40.1 | 0.18927739 | 1 | 0.06309294 | 0.08921208 | -0.02611913 |
| PC.ae.C40.4 | 0.33566434 | 1 | 0.11066025 | 0.13988619 | -0.02922594 |
| PC.ae.C40.5 | 0.396892 | 1 | 0.14020605 | 0.15347011 | -0.01326406 |
| PC.ae.C40.6 | 0.396892 | 1 | 0.14591329 | 0.20564047 | -0.05972718 |
| PC.ae.C42.5 | 0.69432789 | 1 | 0.11155795 | 0.11327933 | -0.00172138 |
| PC.ae.C42.6 | 0.53581974 | 1 | 0.05537378 | 0.08997063 | -0.03459685 |
| SM.a.C32.1 | 0.12059052 | 1 | 0.35515293 | 0.40996703 | -0.05481409 |
| SM.a.C32.2 | 0.09386169 | 1 | 0.0357537 | 0.05308134 | -0.01732765 |
| SM.a.C33.1 | 0.23185703 | 1 | 0.24757959 | 0.37696106 | -0.12938147 |
| SM.a.C34.1 | 0.396892 | 1 | 5.17982781 | 6.26806515 | -1.08823733 |
| SM.a.C34.2 | 0.396892 | 1 | 0.81431383 | 1.0725877 | -0.25827387 |
| SM.a.C35.1 | 0.23185703 | 1 | 0.15249197 | 0.21517455 | -0.06268258 |
| SM.a.C36.1 | 0.15198135 | 1 | 1.05039838 | 1.57328842 | -0.52289004 |
| SM.a.C36.2 | 0.12059052 | 1 | 0.42066557 | 0.69336259 | -0.27269702 |
| SM.a.C36.3 | 0.01398601 | 0.99300699 | 0.02543982 | 0.05180406 | -0.02636424 |
| SM.a.C38.1 | 0.69432789 | 1 | 1.94825935 | 1.87018722 | 0.07807213 |
| SM.a.C38.2 | 0.28096348 | 1 | 1.0120662 | 1.30687267 | -0.29480647 |
| SM.a.C39.1 | 0.53581974 | 1 | 0.27788783 | 0.32236122 | -0.0444734 |
| SM.a.C40.2 | 0.61258741 | 1 | 1.60038091 | 1.73170454 | -0.13132363 |
| SM.a.C40.3 | 0.86651127 | 1 | 0.43772086 | 0.38981053 | 0.04791033 |
| SM.a.C40.4 | 0.23185703 | 1 | 0.00377884 | 0.00596272 | -0.00218388 |
| SM.a.C41.1 | 0.23185703 | 1 | 0.59673514 | 0.79875378 | -0.20201864 |
| SM.a.C41.2 | 0.396892 | 1 | 0.57023676 | 0.65949069 | -0.08925393 |
| SM.a.C42.1 | 0.61258741 | 1 | 0.96323911 | 1.2635737 | -0.30033459 |
| SM.a.C42.2 | 0.33566434 | 1 | 2.8202344 | 3.41634391 | -0.59610951 |
| SM.a.C42.3 | 0.95508936 | 1 | 1.68496691 | 1.5878908 | 0.09707611 |
| SM.a.C42.4 | 0.46340326 | 1 | 0.38610567 | 0.53504702 | -0.14894135 |
| SM.a.C42.6 | 0.28096348 | 1 | 0.16084572 | 0.2315404 | -0.07069468 |
| SM.a.C33.3 | 0.396892 | 1 | 0.00571042 | 0.00643068 | -0.00072026 |
| SM.a.C37.3 | 0.86651127 | 1 | 0.01455144 | 0.01234604 | 0.0022054 |

# Supplement 2.1: Scatterplots of Metabolite percentage and BMI for HDL

# Supplement 2.2: Scatterplots of Metabolite percentage and BMI for LDL

# Supplement 2.3: Scatterplots of Metabolite percentage and BMI for VLDL

# Supplement 3.1: Results of the Wilcoxon Mann Whitney U Test for phospholipid differences between LDL and HDL

| **Analytes** | **P-Value** | **Bonferroni P-Value** | **Mean LDL** | **Mean HDL** | **Mean LDL - Mean HDL** |
| --- | --- | --- | --- | --- | --- |
| PC.aa.C30.0 | 0.040677102 | 1 | 0.166909245 | 0.126270606 | 0.040638639 |
| PC.aa.C32.0 | 0.012753181 | 0.905475826 | 0.64228647 | 0.729727359 | -0.087440889 |
| PC.aa.C32.1 | 0.834990497 | 1 | 0.579492496 | 0.580076758 | -0.000584261 |
| PC.aa.C34.1 | 0.549927683 | 1 | 9.47912416 | 9.812143718 | -0.333019558 |
| PC.aa.C34.2 | 0.336307407 | 1 | 20.22100641 | 21.01724095 | -0.796234541 |
| PC.aa.C34.3 | 0.001379486 | 0.097943479 | 0.380745797 | 0.475036047 | -0.09429025 |
| PC.aa.C34.4 | 0.184131272 | 1 | 0.051575565 | 0.059368586 | -0.007793021 |
| PC.aa.C36.1 | 0.006284786 | 0.446219823 | 1.530735002 | 1.776091748 | -0.245356746 |
| PC.aa.C36.2 | 0.38029625 | 1 | 7.659967625 | 7.810930627 | -0.150963002 |
| PC.aa.C36.3 | 0.094834719 | 1 | 6.126436339 | 6.732673522 | -0.606237183 |
| PC.aa.C36.4 | 0.000485465 | 0.034468027 | 8.075064613 | 9.874358501 | -1.799293888 |
| PC.aa.C36.5 | 0.235353631 | 1 | 0.367104821 | 0.349694342 | 0.017410479 |
| PC.aa.C38.0 | 0.68701508 | 1 | 0.13104621 | 0.141310424 | -0.010264214 |
| PC.aa.C38.3 | 0.336307407 | 1 | 1.915010006 | 2.066090284 | -0.151080278 |
| PC.aa.C38.4 | 0.006284786 | 0.446219823 | 3.345975222 | 4.081385688 | -0.735410467 |
| PC.aa.C38.5 | 6.54467E-07 | 4.64672E-05 | 1.302098258 | 1.795716679 | -0.493618421 |
| PC.aa.C38.6 | 0.050071637 | 1 | 3.610588091 | 4.093887134 | -0.483299043 |
| PC.aa.C40.4 | 0.003901812 | 0.277028652 | 0.158655845 | 0.207002031 | -0.048346186 |
| PC.aa.C40.5 | 0.046760813 | 1 | 0.33612532 | 0.411680486 | -0.075555166 |
| PC.aa.C40.6 | 0.513330159 | 1 | 1.039517833 | 1.103622401 | -0.064104569 |
| PC.ae.C44.6 | 0.005210852 | 0.369970496 | 0.124869165 | 0.205678871 | -0.080809706 |
| PC.ae.C44.5 | 0.000334203 | 0.023728411 | 0.025221344 | 0.041729682 | -0.016508338 |
| PC.ae.C32.0 | 0.174937387 | 1 | 0.119799164 | 0.109036515 | 0.010762649 |
| PC.ae.C32.1 | 0.000485465 | 0.034468027 | 0.097528513 | 0.06959837 | 0.027930143 |
| PC.ae.C32.2 | 0.395693036 | 1 | 0.017965078 | 0.01696676 | 0.000998318 |
| PC.ae.C34.0 | 0.157561087 | 1 | 0.049425151 | 0.060327858 | -0.010902707 |
| PC.ae.C34.1 | 0.106728355 | 1 | 0.318254903 | 0.345517374 | -0.027262471 |
| PC.ae.C34.2 | 0.878599004 | 1 | 0.374749939 | 0.370026669 | 0.00472327 |
| PC.ae.C34.3 | 0.770535421 | 1 | 0.216623852 | 0.219595515 | -0.002971664 |
| PC.ae.C36.1 | 0.16608251 | 1 | 0.184280774 | 0.211203864 | -0.02692309 |
| PC.ae.C36.2 | 0.295660606 | 1 | 0.487229263 | 0.527058646 | -0.039829383 |
| PC.ae.C36.3 | 0.000151954 | 0.010788738 | 0.259182062 | 0.337286628 | -0.078104566 |
| PC.ae.C36.4 | 0.001234907 | 0.087678383 | 0.47602542 | 0.619659101 | -0.143633681 |
| PC.ae.C36.5 | 0.001714881 | 0.121756563 | 0.306171116 | 0.399691451 | -0.093520335 |
| PC.ae.C38.0 | 0.003199365 | 0.227154895 | 0.042150476 | 0.065353426 | -0.02320295 |
| PC.ae.C38.2 | 0.235353631 | 1 | 0.091744134 | 0.082161086 | 0.009583048 |
| PC.ae.C38.3 | 0.478013402 | 1 | 0.148042573 | 0.150762696 | -0.002720123 |
| PC.ae.C38.4 | 0.001234907 | 0.087678383 | 0.418213944 | 0.520986627 | -0.102772682 |
| PC.ae.C38.5 | 2.23234E-06 | 0.000158496 | 0.576833903 | 0.820078191 | -0.243244288 |
| PC.ae.C38.6 | 0.043631742 | 1 | 0.222082565 | 0.263099141 | -0.041016576 |
| PC.ae.C40.0 | 0.009026532 | 0.640883793 | 0.518021648 | 0.701649362 | -0.183627714 |
| PC.ae.C40.1 | 0.017735971 | 1 | 0.04485886 | 0.064813875 | -0.019955014 |
| PC.ae.C40.4 | 0.001908454 | 0.135500206 | 0.0993676 | 0.129934589 | -0.03056699 |
| PC.ae.C40.5 | 0.00572577 | 0.406529689 | 0.126902326 | 0.168405343 | -0.041503017 |
| PC.ae.C40.6 | 0.000258613 | 0.018361525 | 0.135505775 | 0.184574062 | -0.049068287 |
| PC.ae.C42.5 | 0.074172488 | 1 | 0.106345653 | 0.127287934 | -0.020942281 |
| PC.ae.C42.6 | 0.001908454 | 0.135500206 | 0.075614787 | 0.115626287 | -0.040011499 |
| SM.a.C32.1 | 1.0162E-07 | 7.21504E-06 | 0.420827187 | 0.246495732 | 0.174331455 |
| SM.a.C32.2 | 0.141495197 | 1 | 0.030814418 | 0.024385832 | 0.006428585 |
| SM.a.C33.1 | 2.23234E-06 | 0.000158496 | 0.314869275 | 0.188724386 | 0.126144888 |
| SM.a.C34.1 | 8.70533E-10 | 6.18079E-08 | 7.16580359 | 4.4045236 | 2.76127999 |
| SM.a.C34.2 | 2.60358E-05 | 0.001848544 | 1.043705167 | 0.842104764 | 0.201600402 |
| SM.a.C35.1 | 1.83506E-06 | 0.000130289 | 0.201020672 | 0.114492991 | 0.086527681 |
| SM.a.C36.1 | 2.90178E-11 | 2.06026E-09 | 1.603607837 | 0.976768705 | 0.626839132 |
| SM.a.C36.2 | 0.003535193 | 0.250998738 | 0.622652978 | 0.516397842 | 0.106255136 |
| SM.a.C36.3 | 0.495507693 | 1 | 0.036577073 | 0.039817618 | -0.003240545 |
| SM.a.C38.1 | 0.017735971 | 1 | 1.561576196 | 1.286952309 | 0.274623887 |
| SM.a.C38.2 | 0.074172488 | 1 | 0.911671602 | 0.976415824 | -0.064744221 |
| SM.a.C39.1 | 4.74513E-06 | 0.000336904 | 0.351061522 | 0.198761713 | 0.152299809 |
| SM.a.C40.2 | 0.040677102 | 1 | 1.480258233 | 1.316791606 | 0.163466628 |
| SM.a.C40.3 | 0.084003076 | 1 | 0.427075878 | 0.502082449 | -0.075006571 |
| SM.a.C40.4 | 0.126690025 | 1 | 0.003138144 | 0.003517253 | -0.000379108 |
| SM.a.C41.1 | 1.6662E-07 | 1.183E-05 | 1.212244001 | 0.726871449 | 0.485372552 |
| SM.a.C41.2 | 0.001908454 | 0.135500206 | 0.784626592 | 0.581300223 | 0.203326369 |
| SM.a.C42.1 | 0.00011533 | 0.008188451 | 1.823005039 | 1.366478797 | 0.456526242 |
| SM.a.C42.2 | 8.10234E-07 | 5.75266E-05 | 4.824017356 | 3.616754752 | 1.207262603 |
| SM.a.C42.3 | 1.0162E-07 | 7.21504E-06 | 1.957827198 | 1.484499712 | 0.473327485 |
| SM.a.C42.4 | 0.336307407 | 1 | 0.287192488 | 0.246758707 | 0.040433782 |
| SM.a.C42.6 | 0.119743643 | 1 | 0.132872616 | 0.152087694 | -0.019215078 |
| SM.a.C33.3 | 4.83208E-05 | 0.00343078 | 0.009808229 | 0.005605726 | 0.004202503 |
| SM.a.C37.3 | 0.065281383 | 1 | 0.011271398 | 0.008992504 | 0.002278895 |

# Supplement 3.2: Results of the Wilcoxon Mann Whitney U Test for phospholipid differences between VLDL and HDL

| **Analytes** | **P-Value** | **Bonferroni P-Value** | **Mean_VLDL** | **Mean_HDL** | **Mean VLDL - Mean HDL** |
| --- | --- | --- | --- | --- | --- |
| PC.aa.C30.0 | 0.2709887 | 1 | 0.14894892 | 0.12627061 | 0.022678317 |
| PC.aa.C32.0 | 0.00015637 | 0.011102131 | 0.53995963 | 0.72972736 | -0.189767733 |
| PC.aa.C32.1 | 0.03017729 | 1 | 0.82242862 | 0.58007676 | 0.242351866 |
| PC.aa.C34.1 | 0.02501704 | 1 | 11.3461922 | 9.81214372 | 1.534048458 |
| PC.aa.C34.2 | 0.00566573 | 0.402266874 | 23.7249292 | 21.0172409 | 2.707688287 |
| PC.aa.C34.3 | 0.83733425 | 1 | 0.46651256 | 0.47503605 | -0.008523488 |
| PC.aa.C34.4 | 0.22808104 | 1 | 0.055796 | 0.05936859 | -0.00357259 |
| PC.aa.C36.1 | 0.37201771 | 1 | 1.70857815 | 1.77609175 | -0.067513601 |
| PC.aa.C36.2 | 0.01867658 | 1 | 8.71680929 | 7.81093063 | 0.905878663 |
| PC.aa.C36.3 | 0.9182506 | 1 | 6.64606374 | 6.73267352 | -0.086609778 |
| PC.aa.C36.4 | 0.53732959 | 1 | 9.47150754 | 9.8743585 | -0.402850957 |
| PC.aa.C36.5 | 0.53732959 | 1 | 0.32703887 | 0.34969434 | -0.022655473 |
| PC.aa.C38.0 | 0.2709887 | 1 | 0.12639171 | 0.14131042 | -0.014918715 |
| PC.aa.C38.3 | 0.1195561 | 1 | 1.80982225 | 2.06609028 | -0.256268031 |
| PC.aa.C38.4 | 0.9182506 | 1 | 4.05093477 | 4.08138569 | -0.030450921 |
| PC.aa.C38.5 | 0.00270579 | 0.192111 | 1.52272554 | 1.79571668 | -0.272991141 |
| PC.aa.C38.6 | 0.41017444 | 1 | 4.39376836 | 4.09388713 | 0.299881225 |
| PC.aa.C40.4 | 0.00078012 | 0.055388238 | 0.13366513 | 0.20700203 | -0.073336904 |
| PC.aa.C40.5 | 0.33608391 | 1 | 0.36418968 | 0.41168049 | -0.047490802 |
| PC.aa.C40.6 | 0.63161845 | 1 | 1.15874647 | 1.1036224 | 0.055124072 |
| PC.ae.C44.6 | 0.00026047 | 0.018493069 | 0.0933052 | 0.20567887 | -0.112373672 |
| PC.ae.C44.5 | 2.8486E-05 | 0.002022541 | 0.01629683 | 0.04172968 | -0.025432848 |
| PC.ae.C32.0 | 0.02061852 | 1 | 0.09285215 | 0.10903652 | -0.016184363 |
| PC.ae.C32.1 | 0.83733425 | 1 | 0.07228166 | 0.06959837 | 0.002683288 |
| PC.ae.C32.2 | 0.06043639 | 1 | 0.02679102 | 0.01696676 | 0.009824256 |
| PC.ae.C34.0 | 0.02749607 | 1 | 0.04270277 | 0.06032786 | -0.017625088 |
| PC.ae.C34.1 | 0.68118707 | 1 | 0.33444511 | 0.34551737 | -0.011072261 |
| PC.ae.C34.2 | 0.20223637 | 1 | 0.34278075 | 0.37002667 | -0.027245917 |
| PC.ae.C34.3 | 0.68118707 | 1 | 0.20885492 | 0.21959552 | -0.010740599 |
| PC.ae.C36.1 | 0.53732959 | 1 | 0.22044084 | 0.21120386 | 0.009236978 |
| PC.ae.C36.2 | 0.9454487 | 1 | 0.54267707 | 0.52705865 | 0.015618423 |
| PC.ae.C36.3 | 0.00013114 | 0.009311186 | 0.22922707 | 0.33728663 | -0.108059562 |
| PC.ae.C36.4 | 0.0227283 | 1 | 0.4995987 | 0.6196591 | -0.120060402 |
| PC.ae.C36.5 | 0.30240435 | 1 | 0.36201233 | 0.39969145 | -0.037679126 |
| PC.ae.C38.0 | 0.43007046 | 1 | 0.05984783 | 0.06535343 | -0.005505594 |
| PC.ae.C38.2 | 0.78425382 | 1 | 0.07953265 | 0.08216109 | -0.002628436 |
| PC.ae.C38.3 | 0.00566573 | 0.402266874 | 0.09942542 | 0.1507627 | -0.051337272 |
| PC.ae.C38.4 | 6.2852E-05 | 0.00446252 | 0.36858108 | 0.52098663 | -0.152405544 |
| PC.ae.C38.5 | 4.0195E-07 | 2.85382E-05 | 0.51745442 | 0.82007819 | -0.302623766 |
| PC.ae.C38.6 | 0.24182613 | 1 | 0.23142423 | 0.26309914 | -0.031674915 |
| PC.ae.C40.0 | 0.41017444 | 1 | 0.63571482 | 0.70164936 | -0.065934547 |
| PC.ae.C40.1 | 0.09630051 | 1 | 0.05130294 | 0.06481387 | -0.013510937 |
| PC.ae.C40.4 | 0.00159027 | 0.11290942 | 0.0897455 | 0.12993459 | -0.040189085 |
| PC.ae.C40.5 | 0.00159027 | 0.11290942 | 0.10620493 | 0.16840534 | -0.062200412 |
| PC.ae.C40.6 | 9.1385E-05 | 0.006488351 | 0.12110552 | 0.18457406 | -0.063468545 |
| PC.ae.C42.5 | 0.00348766 | 0.247624102 | 0.09251109 | 0.12728793 | -0.03477684 |
| PC.ae.C42.6 | 9.6629E-06 | 0.000686065 | 0.05666029 | 0.11562629 | -0.058966 |
| SM.a.C32.1 | 0.02501704 | 1 | 0.29034222 | 0.24649573 | 0.043846489 |
| SM.a.C32.2 | 0.00566573 | 0.402266874 | 0.03866354 | 0.02438583 | 0.014277712 |
| SM.a.C33.1 | 0.01375419 | 0.976547647 | 0.22798878 | 0.18872439 | 0.039264398 |
| SM.a.C34.1 | 0.02749607 | 1 | 4.14010418 | 4.4045236 | -0.264419425 |
| SM.a.C34.2 | 0.03307281 | 1 | 0.72731646 | 0.84210476 | -0.114788306 |
| SM.a.C35.1 | 0.53732959 | 1 | 0.12203414 | 0.11449299 | 0.007541153 |
| SM.a.C36.1 | 0.4505003 | 1 | 0.92685579 | 0.97676871 | -0.049912915 |
| SM.a.C36.2 | 0.00800135 | 0.568095555 | 0.41015531 | 0.51639784 | -0.106242536 |
| SM.a.C36.3 | 0.43007046 | 1 | 0.03507941 | 0.03981762 | -0.004738212 |
| SM.a.C38.1 | 0.97270883 | 1 | 1.29105792 | 1.28695231 | 0.00410561 |
| SM.a.C38.2 | 0.00446155 | 0.316769978 | 0.86585298 | 0.97641582 | -0.110562839 |
| SM.a.C39.1 | 0.53732959 | 1 | 0.25560026 | 0.19876171 | 0.056838544 |
| SM.a.C40.2 | 0.56024497 | 1 | 1.28713179 | 1.31679161 | -0.029659812 |
| SM.a.C40.3 | 0.0767222 | 1 | 0.3899825 | 0.50208245 | -0.112099951 |
| SM.a.C40.4 | 0.60740877 | 1 | 0.00360883 | 0.00351725 | 9.15805E-05 |
| SM.a.C41.1 | 0.00026047 | 0.018493069 | 0.48193197 | 0.72687145 | -0.244939479 |
| SM.a.C41.2 | 0.00894638 | 0.6351928 | 0.44811954 | 0.58130022 | -0.13318068 |
| SM.a.C42.1 | 1.3028E-06 | 9.25005E-05 | 0.78301574 | 1.3664788 | -0.583463057 |
| SM.a.C42.2 | 1.4979E-07 | 1.06349E-05 | 2.30258483 | 3.61675475 | -1.314169924 |
| SM.a.C42.3 | 0.03955745 | 1 | 1.23383949 | 1.48449971 | -0.250660221 |
| SM.a.C42.4 | 0.03955745 | 1 | 0.4542757 | 0.24675871 | 0.20751699 |
| SM.a.C42.6 | 0.33608391 | 1 | 0.14087016 | 0.15208769 | -0.011217532 |
| SM.a.C33.3 | 0.35377044 | 1 | 0.00487541 | 0.00560573 | -0.000730312 |
| SM.a.C37.3 | 0.73213266 | 1 | 0.00992726 | 0.0089925 | 0.000934758 |

# Supplement 3.2: Results of the Wilcoxon Mann Whitney U Test for phospholipid differences between VLDL and LDL

| **Analytes** | **P-Value** | **Bonferroni P-Value** | **Mean VLDL** | **Mean LDL** | **Mean VLDL - Mean LDL** |
| --- | --- | --- | --- | --- | --- |
| PC.aa.C30.0 | 0.38185661 | 1 | 0.14894892 | 0.16690924 | -0.017960322 |
| PC.aa.C32.0 | 0.00237729 | 0.16878788 | 0.53995963 | 0.64228647 | -0.102326844 |
| PC.aa.C32.1 | 0.01734911 | 1 | 0.82242862 | 0.5794925 | 0.242936127 |
| PC.aa.C34.1 | 0.00547708 | 0.38887241 | 11.3461922 | 9.47912416 | 1.867068016 |
| PC.aa.C34.2 | 0.00161861 | 0.1149215 | 23.7249292 | 20.2210064 | 3.503922829 |
| PC.aa.C34.3 | 0.00386965 | 0.27474504 | 0.46651256 | 0.3807458 | 0.085766762 |
| PC.aa.C34.4 | 0.90835568 | 1 | 0.055796 | 0.05157557 | 0.004220431 |
| PC.aa.C36.1 | 0.21431612 | 1 | 1.70857815 | 1.530735 | 0.177843145 |
| PC.aa.C36.2 | 0.00547708 | 0.38887241 | 8.71680929 | 7.65996762 | 1.056841666 |
| PC.aa.C36.3 | 0.06887068 | 1 | 6.64606374 | 6.12643634 | 0.519627405 |
| PC.aa.C36.4 | 0.00946228 | 0.67182193 | 9.47150754 | 8.07506461 | 1.396442931 |
| PC.aa.C36.5 | 0.4190446 | 1 | 0.32703887 | 0.36710482 | -0.040065951 |
| PC.aa.C38.0 | 0.52056212 | 1 | 0.12639171 | 0.13104621 | -0.004654501 |
| PC.aa.C38.3 | 0.6331133 | 1 | 1.80982225 | 1.91501001 | -0.105187753 |
| PC.aa.C38.4 | 0.01573863 | 1 | 4.05093477 | 3.34597522 | 0.704959546 |
| PC.aa.C38.5 | 0.01050687 | 0.74598802 | 1.52272554 | 1.30209826 | 0.22062728 |
| PC.aa.C38.6 | 0.01573863 | 1 | 4.39376836 | 3.61058809 | 0.783180269 |
| PC.aa.C40.4 | 0.31364486 | 1 | 0.13366513 | 0.15865585 | -0.024990719 |
| PC.aa.C40.5 | 0.45822364 | 1 | 0.36418968 | 0.33612532 | 0.028064364 |
| PC.aa.C40.6 | 0.24011456 | 1 | 1.15874647 | 1.03951783 | 0.119228641 |
| PC.ae.C44.6 | 0.13059441 | 1 | 0.0933052 | 0.12486917 | -0.031563966 |
| PC.ae.C44.5 | 0.01909764 | 1 | 0.01629683 | 0.02522134 | -0.008924511 |
| PC.ae.C32.0 | 0.00488626 | 0.34692457 | 0.09285215 | 0.11979916 | -0.026947012 |
| PC.ae.C32.1 | 0.00946228 | 0.67182193 | 0.07228166 | 0.09752851 | -0.025246855 |
| PC.ae.C32.2 | 0.09937712 | 1 | 0.02679102 | 0.01796508 | 0.008825938 |
| PC.ae.C34.0 | 0.22696112 | 1 | 0.04270277 | 0.04942515 | -0.00672238 |
| PC.ae.C34.1 | 0.65677594 | 1 | 0.33444511 | 0.3182549 | 0.016190209 |
| PC.ae.C34.2 | 0.16869017 | 1 | 0.34278075 | 0.37474994 | -0.031969187 |
| PC.ae.C34.3 | 1 | 1 | 0.20885492 | 0.21662385 | -0.007768936 |
| PC.ae.C36.1 | 0.06887068 | 1 | 0.22044084 | 0.18428077 | 0.036160068 |
| PC.ae.C36.2 | 0.26796494 | 1 | 0.54267707 | 0.48722926 | 0.055447806 |
| PC.ae.C36.3 | 0.19052671 | 1 | 0.22922707 | 0.25918206 | -0.029954996 |
| PC.ae.C36.4 | 0.45822364 | 1 | 0.4995987 | 0.47602542 | 0.023573279 |
| PC.ae.C36.5 | 0.07428214 | 1 | 0.36201233 | 0.30617112 | 0.055841209 |
| PC.ae.C38.0 | 0.02765924 | 1 | 0.05984783 | 0.04215048 | 0.017697356 |
| PC.ae.C38.2 | 0.45822364 | 1 | 0.07953265 | 0.09174413 | -0.012211484 |
| PC.ae.C38.3 | 0.00946228 | 0.67182193 | 0.09942542 | 0.14804257 | -0.048617149 |
| PC.ae.C38.4 | 0.07428214 | 1 | 0.36858108 | 0.41821394 | -0.049632861 |
| PC.ae.C38.5 | 0.28266892 | 1 | 0.51745442 | 0.5768339 | -0.059379478 |
| PC.ae.C38.6 | 0.60981073 | 1 | 0.23142423 | 0.22208257 | 0.009341661 |
| PC.ae.C40.0 | 0.25378106 | 1 | 0.63571482 | 0.51802165 | 0.117693168 |
| PC.ae.C40.1 | 0.80512309 | 1 | 0.05130294 | 0.04485886 | 0.006444078 |
| PC.ae.C40.4 | 0.3299177 | 1 | 0.0897455 | 0.0993676 | -0.009622095 |
| PC.ae.C40.5 | 0.13059441 | 1 | 0.10620493 | 0.12690233 | -0.020697395 |
| PC.ae.C40.6 | 0.4993215 | 1 | 0.12110552 | 0.13550577 | -0.014400258 |
| PC.ae.C42.5 | 0.13944577 | 1 | 0.09251109 | 0.10634565 | -0.013834559 |
| PC.ae.C42.6 | 0.05450123 | 1 | 0.05666029 | 0.07561479 | -0.018954501 |
| SM.a.C32.1 | 9.0622E-05 | 0.00643418 | 0.29034222 | 0.42082719 | -0.130484966 |
| SM.a.C32.2 | 0.07428214 | 1 | 0.03866354 | 0.03081442 | 0.007849127 |
| SM.a.C33.1 | 0.00033842 | 0.02402811 | 0.22798878 | 0.31486927 | -0.086880491 |
| SM.a.C34.1 | 1.8473E-08 | 1.3116E-06 | 4.14010418 | 7.16580359 | -3.025699415 |
| SM.a.C34.2 | 1.2765E-06 | 9.0632E-05 | 0.72731646 | 1.04370517 | -0.316388708 |
| SM.a.C35.1 | 0.00045875 | 0.03257147 | 0.12203414 | 0.20102067 | -0.078986529 |
| SM.a.C36.1 | 2.4631E-09 | 1.7488E-07 | 0.92685579 | 1.60360784 | -0.676752047 |
| SM.a.C36.2 | 8.651E-06 | 0.00061422 | 0.41015531 | 0.62265298 | -0.212497671 |
| SM.a.C36.3 | 0.34671227 | 1 | 0.03507941 | 0.03657707 | -0.001497667 |
| SM.a.C38.1 | 0.14873867 | 1 | 1.29105792 | 1.5615762 | -0.270518277 |
| SM.a.C38.2 | 0.24011456 | 1 | 0.86585298 | 0.9116716 | -0.045818618 |
| SM.a.C39.1 | 0.00161861 | 0.1149215 | 0.25560026 | 0.35106152 | -0.095461266 |
| SM.a.C40.2 | 0.03302169 | 1 | 1.28713179 | 1.48025823 | -0.19312644 |
| SM.a.C40.3 | 0.31364486 | 1 | 0.3899825 | 0.42707588 | -0.03709338 |
| SM.a.C40.4 | 0.1065766 | 1 | 0.00360883 | 0.00313814 | 0.000470689 |
| SM.a.C41.1 | 4.3104E-09 | 3.0604E-07 | 0.48193197 | 1.212244 | -0.730312031 |
| SM.a.C41.2 | 2.7279E-06 | 0.00019368 | 0.44811954 | 0.78462659 | -0.336507049 |
| SM.a.C42.1 | 2.771E-08 | 1.9674E-06 | 0.78301574 | 1.82300504 | -1.039989299 |
| SM.a.C42.2 | 1.2315E-09 | 8.744E-08 | 2.30258483 | 4.82401736 | -2.521432527 |
| SM.a.C42.3 | 2.4528E-05 | 0.00174145 | 1.23383949 | 1.9578272 | -0.723987707 |
| SM.a.C42.4 | 0.20217343 | 1 | 0.4542757 | 0.28719249 | 0.167083209 |
| SM.a.C42.6 | 0.70509966 | 1 | 0.14087016 | 0.13287262 | 0.007997546 |
| SM.a.C33.3 | 1.6364E-05 | 0.00116181 | 0.00487541 | 0.00980823 | -0.004932815 |
| SM.a.C37.3 | 0.43838939 | 1 | 0.00992726 | 0.0112714 | -0.001344137 |

# Supplement 4.1: Scatterplot for the association of phospholipid species and cholesterol in HDL

# Supplement 4.2: Scatterplot for the association of phospholipid species and cholesterol in LDL
